# The gene regulatory network of *Staphylococcus aureus* ST239-SCC*mec*III strain Bmb9393 and assessment of genes associated with the biofilm in diverse backgrounds

**DOI:** 10.3389/fmicb.2022.1049819

**Published:** 2023-01-10

**Authors:** Maiana de Oliveira Cerqueira e Costa, Ana Paula Barbosa do Nascimento, Yasmmin Cortes Martins, Marcelo Trindade dos Santos, Agnes Marie de Sá Figueiredo, Ernesto Perez-Rueda, Marisa Fabiana Nicolás

**Affiliations:** ^1^Laboratório Nacional de Computação Científica (LNCC), Petrópolis, Brazil; ^2^Departamento de Análises Clínicas e Toxicológicas, Faculdade de Ciências Farmacêuticas, Universidade de São Paulo, São Paulo, Brazil; ^3^Instituto de Investigaciones en Matemáticas Aplicadas y en Sistemas, Universidad Nacional Autónoma de México, Unidad Académica Yucatán, Merida, Mexico; ^4^Laboratório de Biologia Molecular de Bactérias, Instituto de Microbiologia Paulo de Goés, Universidade Federal do Rio de Janeiro, Rio de Janeiro, Brazil

**Keywords:** *Staphylococcus aureus*, gene regulatory network, biofilm, motifs, transcriptional regulatory network, computational model

## Abstract

**Introduction:**

*Staphylococcus aureus* is one of the most prevalent and relevant pathogens responsible for a wide spectrum of hospital-associated or community-acquired infections. In addition, methicillin-resistant *Staphylococcus aureus* may display multidrug resistance profiles that complicate treatment and increase the mortality rate. The ability to produce biofilm, particularly in device-associated infections, promotes chronic and potentially more severe infections originating from the primary site. Understanding the complex mechanisms involved in planktonic and biofilm growth is critical to identifying regulatory connections and ways to overcome the global health problem of multidrug-resistant bacteria.

**Methods:**

In this work, we apply literature-based and comparative genomics approaches to reconstruct the gene regulatory network of the high biofilm-producing strain Bmb9393, belonging to one of the highly disseminating successful clones, the Brazilian epidemic clone. To the best of our knowledge, we describe for the first time the topological properties and network motifs for the *Staphylococcus aureus* pathogen. We performed this analysis using the ST239-SCC*mec*III Bmb9393 strain. In addition, we analyzed transcriptomes available in the literature to construct a set of genes differentially expressed in the biofilm, covering different stages of the biofilms and genetic backgrounds of the strains.

**Results and discussion:**

The Bmb9393 gene regulatory network comprises 1,803 regulatory interactions between 64 transcription factors and the non-redundant set of 1,151 target genes with the inclusion of 19 new regulons compared to the N315 transcriptional regulatory network published in 2011. In the Bmb9393 network, we found 54 feed-forward loop motifs, where the most prevalent were coherent type 2 and incoherent type 2. The non-redundant set of differentially expressed genes in the biofilm consisted of 1,794 genes with functional categories relevant for adaptation to the variable microenvironments established throughout the biofilm formation process. Finally, we mapped the set of genes with altered expression in the biofilm in the Bmb9393 gene regulatory network to depict how different growth modes can alter the regulatory systems. The data revealed 45 transcription factors and 876 shared target genes. Thus, the gene regulatory network model provided represents the most up-to-date model for *Staphylococcus aureus*, and the set of genes altered in the biofilm provides a global view of their influence on biofilm formation from distinct experimental perspectives and different strain backgrounds.

## 1. Introduction

Methicillin-resistant *Staphylococcus aureus* is a Gram-positive bacterium that causes a broad range of hospital infections contributing to increased morbidity and mortality. The presence of an extensive and diverse virulence repertoire, in addition to notable antimicrobial multiresistance phenotypes, make *S. aureus* one of the main public health concerns worldwide. A very alarming statistic unveiled by Bardi et al. (2021) was the increase of primary and secondary infections related to medical devices such as catheters and the incidence of pneumonia in patients with severe COVID-19 admitted to intensive care units, where *S. aureus* was one of the main causative agents in both situations. Bacterial biofilm growth provides advantages compared to the planktonic lifestyle, and it is an important mechanism of chronic infections, often found in medical devices (Figueiredo et al., 2017). Biofilm formation involves four main stages: attachment, proliferation, maturation, and detachment (Schilcher and Horswill, 2020). Regarding the composition, biofilm varies according to the strain and can be classified into *ica*-dependent and independent. The biofilm formation *ica*-dependent is mediated by polysaccharide intercellular adhesin (PIA), and is more common in methicillin-susceptible *S. aureus* (MSSA). The biofilm formation *ica*-independent is more common in methicillin-resistant *S. aureus* (MRSA), and the multifactorial mechanisms involved are not fully understood (O'Neill et al., 2007; Schilcher and Horswill, 2020). In addition, the same strain can produce different types of biofilm matrix depending on environmental cues (Figueiredo et al., 2017; Schilcher and Horswill, 2020).

Responses to environmental changes are triggered by modifications in the gene expression patterns. The most common modification occurs at the transcriptional level controlled by the transcription factors (TFs). TFs recognize conserved nucleotide sequences in the promoter region of target genes (TGs) called transcription factor binding sites (TFBSs), thus controlling the gene expression according to the environmental changes (Mercatelli et al., 2020). The set of genes or operons regulated directly by the same TF is defined as a regulon, and the set of regulons constitutes the gene regulatory network (GRN) of an organism (Rodionov, 2007). In Systems Biology, the set of regulatory interactions between TFs and their target genes can be modeled as a directed graph, where regulators and targets are nodes, and interactions are edges whose direction depicts the information flow (TF → TG) (Barabási and Oltvai, 2004). A GRN can be decomposed into subgraphs of recurrent regulatory patterns called network motifs that act to define the regulatory state of a target gene (Alon, 2007; Stone et al., 2019). It is possible to obtain relevant information from biological networks by exploring their topological metrics and properties from graph theory.

GRN inference provides a systematic comprehension of molecular mechanisms that regulate metabolic phenotypes of organisms under distinct endogenous or environmental conditions. The methods of GRN reconstruction are diverse and vary according to the input data and prediction goal. Faria et al. (2014) proposed the classification of the methods as genomic-driven and data-driven, the first is based on comparative genomics approaches, and the latter is based on reverse engineering from expression data (Faria et al., 2014). A recent review of different GRN inference methodologies can be found in the work of Mercatelli et al. (2020). The work published by Ravcheev et al. (2011) applied the genomic-driven method implemented in the RegPredict web server to infer the first GRN for *S. aureus* strain N315, an intermediate biofilm-producer belonging to the clonotype II-A (SCC*mec*II), and other six species of the *Staphylococcaceae* family (Ravcheev et al., 2011). However, N315 GRN has not been updated since 2011, and several regulons essential to biofilm formation, virulence, and antimicrobial resistance are absent.

In our work, we reconstructed a GRN for the MRSA strain Bmb9393 by applying comparative genomics and literature-based approaches. Bmb9393 is a high biofilm-producing Brazilian strain possessing a resistance profile to a broad range of antimicrobials. The Bmb9393 genome is the first strain belonging to ST239-SCC*mec*III lineage from Latin America to be available in databases. For the first time, we described the topological characteristics and identified network motifs for an *S. aureus* GRN. In addition, we profiled a genomic landscape of differentially expressed genes (DEGs) in the biofilm condition by analyzing several transcriptomes available in the literature. Finally, we mapped the DEGs into the Bmb9393 GRN to portray how growth in the biofilm can alter the regulatory systems. Thus, our work not only provides an updated GRN based on a strain with a genetic and phenotypic background considerably distinct from the N315 strain but also offers a global view of biofilm formation from the regulatory perspective of different isolates.

## 2. Materials and methods

### 2.1. Identification of the transcription factor repertoire of Bmb9393 strain

The nucleotide and amino acid sequence of the complete genome of Bmb9393 strain were obtained from the NCBI FTP server. The first step toward the identification of the transcription factor (TF) repertoire was the Bmb9393 genome re-annotation, which was based on similarity searches against the UniProtKB/Swiss-Prot database (UniProt Consortium, 2019) using the BLASTp algorithm with an e-value ≤ 10^−5^ and a coverage ≥ 80%. We compared the previous annotation with BLAST results and updated the information accordingly. In addition, to identify the DNA-binding domains, we locally run InterProScan (version 5.39-77.0) with default parameters to map the InterPro families and Pfam assignments of the entire proteome (Jones et al., 2014). Next, we retrieved TF information from databases such as the *Predicted Prokaryotic Transcription Factor* (P2TF), one of the most relevant, because it provided the TF classification into regulatory families (Ortet et al., 2012). Besides, we submitted the Bmb9393 genome to the *Predicted Prokaryotic Regulatory Proteins* (P2RP) tool to identify regulatory proteins (Barakat et al., 2013). The P2TF and P2RP results were compared to the previous annotation, and information was updated accordingly. Then, we searched the literature with specific terms such as “*transcriptional regulator*,” “*response regulator*,” “*activator*,” and “*repressor*” to identify articles associated with new TFs in *S. aureus*. Once a TF was found, we included it in the Bmb9393 annotation after ortholog detection using ProteinOrtho (Lechner et al., 2011). To compare strains of the same species, we used more restrictive parameters for the alignment: e-value ≤ 10^−10^, identity ≥ 35%, and minimum coverage of 80%. Finally, we defined a protein as TF after manually checking the results obtained with BLASTp against UniProtKB/Swiss-Prot, PFAM assignments of DNA-binding domains, and the genes identified by P2TF and P2RP.

### 2.2. Gene regulatory network reconstruction of Bmb9393 strain

The GRN reconstruction of Bmb9393 strain was performed based on a hybrid methodology comprising a reference-based inference and the prediction of *cis*-regulatory elements. The identification of regulatory interactions between TFs and their target genes was obtained by: (i) comparative propagation of the GRN of *S. aureus* strain N315 (Ravcheev et al., 2011); (ii) identification of TFBSs in the upstream region of Bmb9393 coding sequences using the DNA-binding sequences from orthologous TFs of *S. aureus*, and the model Gram-positive bacterium *Bacillus subtilis* 168; and (iii) literature-based inference of regulons. In step (i), we retrieved TFs and target genes and the available TFBSs from the RegPrecise database, including those on the GRN of N315 published in 2011 (Ravcheev et al., 2011; Novichkov et al., 2013). To propagate the network, we detected the set of orthologous TFs and target genes between the N315 and Bmb9393 using the ProteinOrtho (Lechner et al., 2011) with the same parameters described above, thus obtaining the first draft of the GRN of Bmb9393.

In step (ii), we applied the concept of operon-based expansion used in the reconstruction of the GRN of *Mycobacterium tuberculosis* (Sanz et al., 2011). Briefly, if gene A regulates gene B, and gene B is part of the BCD operon, then we included the interactions A → C and A → D in the network. For TFBSs from *S. aureus*, we built a list for each TF with all TFBSs obtained from RegPrecise (Novichkov et al., 2013), as well as the experimentally validated databases CollecTF (Kiliç et al., 2014) and PRODORIC (Dudek and Jahn, 2022). Each file was given as input to the genome-scale-dna-pattern tool of the Regulatory Sequence Analysis Tools (RSAT) software suite (Santana-Garcia et al., 2022). We executed the application locally to search for TFBSs in the upstream region of Bmb9393 coding sequences obtained through the retrieve-sequence tool of RSAT, covering −400 to +50 bases from the start codon with and without the option noorf (prevent overlap with neighbor genes). Then, we filtered the outputs and mapped the results to retrieve the gene identifier and gene product in the Bmb9393 genome, comparing each list of possible target genes with those associated with the same TF already included in the Bmb9393 GRN. If there was a new interaction or regulon, we searched the literature to validate the predicted gene or at least its function as part of that regulon. For TFBSs from *B. subtilis*, we first retrieved a list of TFs from its most recent GRN (Faria et al., 2016) to identify orthologs in the Bmb9393 using ProteinOrtho. To compare different species, we considered the parameters described in Janga and Moreno-Hagelsieb (2004): e-value ≤ 10^−6^ and minimum coverage of 60%. Next, we aligned the TFBSs from the *B. subtilis* orthologous TFs with those recovered from *S. aureus* to quantify the variability between the binding sites according to the classification described in Ravcheev et al. (2011). Only the TFBSs classified as conserved (up to four different bases) were considered when performing the searches in the upstream region of the Bmb9393 coding sequences using the genome-scale-dna-pattern tool. Again, if there was a new interaction or regulon, a manual curation based on literature and databases was the primary method to validate the interactions in *S. aureus*.

In step (iii), we enriched the GRN of Bmb9393 by searching for TFBSs of the new TFs identified in the previous section to infer regulons not yet available in public databases. To this end, we sought in the literature articles from 2010 to 2018 associated with TFBSs for new regulators, since the only *S. aureus* GRN is from 2011, and applied step (ii) for incorporation into the GRN of Bmb9393. After the reconstruction steps, we applied a text mining pipeline to retrieve evidence of transcription factor regulation from the literature using the NCBI PubMed Central API (*Access Programming Interface*) (more details about the pipeline can be found in Supplementary Section 1.1). We used the results obtained from the pipeline to input the potential mode of regulation of a TF upon their targets.

### 2.3. Topological analysis and comparison between the GRNs of Bmb9393 and N315 strains

We performed the network topological analysis using the igraph R package (Csardi and Nepusz, 2006). In addition, to detect *feed-forward loop* (FFL) motifs, we execute the triad_census function extracting the results from the corresponding pattern (more details about the FFL identification can be found in Supplementary Section 1.1) and classifying the types of FFL according to Mangan and Alon (2003). The statistical significance of the motif detection was calculated using the *Z-score* as described in Koutrouli et al. (2020). The GRNs were manipulated with the Cytoscape software (version 3.9.1) (Shannon et al., 2003). We performed the Bmb9393 and N315 network overlapping using DyNet App (Goenawan et al., 2016) for Cytoscape.

### 2.4. Identification of differentially expressed genes in biofilm and overlapping with the GRN of Bmb9393

We obtained the differentially expressed genes (DEGs) from transcriptome analysis of different strains of *S. aureus* cultured under biofilm and planktonic conditions deposited in the former SAMMD database (current SATMD - *Staphylococcus aureus Transcriptome Meta-Database*) (Nagarajan and Elasri, 2007). In addition, we searched the literature on recent *S. aureus* transcriptome studies related to biofilm formation, resulting in the selection of four microarrays and one bulk RNA-seq data (Table 1). We considered for the analysis all DEGs regardless of their direction. This is due to several limiting factors as transcriptomes are from different *S. aureus* strains, collected in distinct timepoints and sequenced with different technologies. We determined one set of non-redundant DEGs from microarrays and another from the bulk RNA-seq (DEGs shared by two strains), and identified the orthologs in the Bmb9393 genome using ProteinOrtho (Lechner et al., 2011). The orthologous DEGs were included in the orthologous set of non-redundant DEGs from microarrays and from RNAseq lists. Next, we retrieved the amino acid sequences of the non-redundant genes from both lists using the SeqKit (Shen et al., 2016) and submitted them to EggNOG-mapper (available online at http://eggnog-mapper.embl.de/) (Huerta-Cepas et al., 2017) to obtain a classification of the DEGs into functional categories of orthologs clusters (COGs) and ontology terms (GO) (Tatusov et al., 2000; The Gene Ontology Consortium, 2019). We used default parameters except for *query* and *subject* minimum coverage of 60%. We used the REVIGO web server (available at http://revigo.irb.hr/) with default parameters to summarize the GO terms obtained from EggNOG-mapper (Supek et al., 2011). In addition, we performed a functional classification of the proteins into KEGG Orthology (KO) groups using BlastKOALA (available at https://www.kegg.jp/blastkoala/) to reconstruct the KEGG pathways using the Reconstruct tool from KEGG Mapper (available at https://www.genome.jp/kegg/mapper/reconstruct.html) (Kanehisa et al., 2016, 2022). Last, we mapped the orthologous set of non-redundant DEGs from microarrays and from RNAseq lists in the GRN of Bmb9393 to recover the regulons containing some or all differentially expressed target genes in biofilm.

**Table 1:** Selection of transcriptome studies of *S. aureus* strains in biofilm and planktonic conditions.

**Techniques**	**Strains**	**DEGs**	**References**
Microarray	UAMS-1	451	Beenken et al., 2004
Microarray	*strain* 113	157	Resch et al., 2005
Microarray	MRSA-M2	20	Brady et al., 2006
Microarray	USA300	471	Stewart et al., 2022
Bulk RNA-seq	N315, MRSA252, LAC, MW2 and NRS385	1310	Tomlinson et al., 2021
DEGs Total		2,409	
Non-redundant DEGs		**1,794**	

The bold value represents the number of non-redundant DEGs among all transcriptome studies.

## 3. Results and discussion

### 3.1. Transcription factor repertoire of the Bmb9393 strain

**Figure 1:**
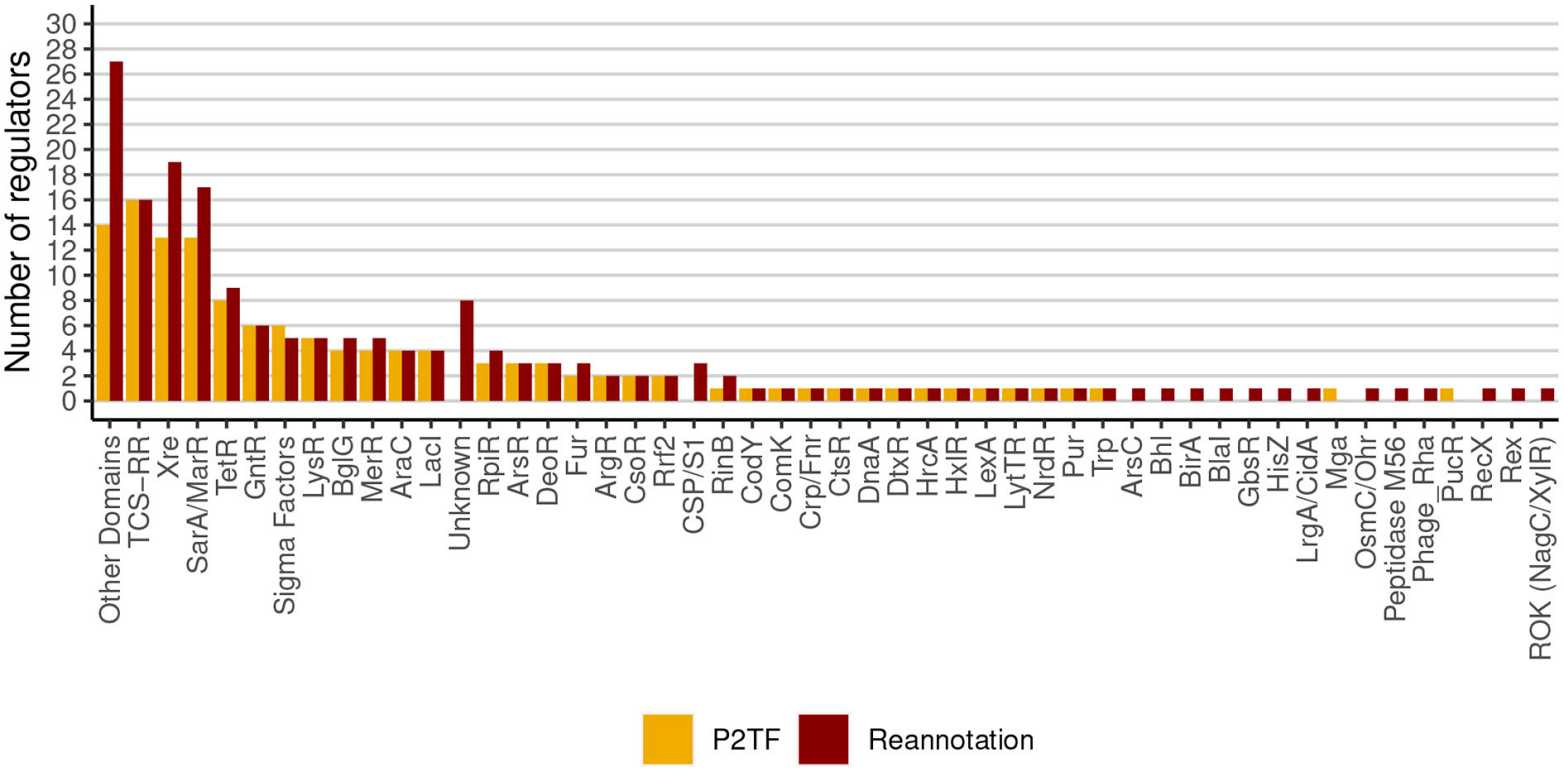
Classification and abundances of regulatory families of Bmb9393. The yellow columns represent the predictions made by the P2TF database, and the red columns represent the TFs repertoire and σ factors obtained from the re-annotation process.

The *S. aureus* strain Bmb9393 is a high biofilm-producing bacterium isolated from nosocomial bacteremia. It is the first strain belonging to the Brazilian epidemic clone (BEC) from the ST239-SCC*mec*III lineage to have its genome fully sequenced (GenBank accession number CP005288 for the chromosome, and CP005289 for the plasmid) (Costa et al., 2013). The re-annotation process of the Bmb9393 genome was important to include new information obtained since the first annotation in 2012. After the re-annotation, we identified a set of non-redundant transcriptional regulators consisting of 175 TFs and five sigma (σ) factors (Supplementary Table S1), classified into 48 regulatory families according to PFAM domains, the P2TF database and Ibarra et al. (2013) (Figure 1). The most represented families in Bmb9393 were Xre with 19 members, and MarR with 17 members, including the Sar proteins, followed by the TCS regulators with 16 members, and TetR family with 9 members. Twenty-seven regulators had domains characterized by PFAM (Finn et al., 2014) but no defined family (*Other Domains*), and 8 regulators had neither defined family nor domain according to PFAM assignments (*Unknown*) (Figure 1). Bmb9393 presented a larger transcription factor repertoire compared with the strains analyzed by Ibarra et al. (2013), whose numbers ranged from 126 to the RF122 bovine strain up to 151 to the N315 strain (more results about the re-annotation process can be found in Supplementary Section 1.2 and Supplementary Figure S1).

Inside the Xre family, *xdrA* (SABB_01966) and *potR* (SABB_01062) are the only genes that have described functions. XdrA is an important transcriptional activator of the staphylococcal protein A (SpA), an important virulence factor (Cruz et al., 2021), whereas PotR is an activator of the *pot*ABCD operon, involved in the polyamine uptake in *S. aureus* (McCallum et al., 2010; Yao and Lu, 2014). Both genes play important roles in pathogenicity and virulence mechanisms, such as biofilm formation, quorum sensing, repression of capsule production, and homeostasis (DeFrancesco et al., 2017; Lei and Lee, 2018; Gimza et al., 2019). Unlike other genes encoding proteins of the Xre family in the Bmb9393 strain, *xdrA* and *potR* are not located inside or near phage-related elements. Of the 21 regulators, 14 are related to regions of genomic plasticity (RGPs), and 5 are conserved among all *S. aureus* strains analyzed by Ibarra et al. (2013): SABB_00224, SABB_02114, SABB_02258 (target gene of CodY), *xdrA* and *potR* (target gene of SrrA and ComK1).

The “Sigma Factors” is the sixth family most represented in the Bmb9393 genome (Figure 1) and includes the four known σ factors of *S. aureus*. While σ^A^ (*sigA* or SABB_00481) controls the expression of most genes, including housekeeping genes, the alternative σ factors regulate subsets of genes involved in specialized functions such as response and adaptation to environmental changes (Paget and Helmann, 2003). In this context, σ^B^ (*sigB* or SABB_02394) plays an important role in the complex transcriptional network of *S. aureus* because it regulates the expression of virulence determinants such as biofilm, and increases the resistance to antimicrobials that target the cell wall (Bischoff et al., 2004; Pané-Farré et al., 2006). In addition, σ^B^ is related to the expression of genes involved with transport and stress response (Ishii et al., 2014; Guldimann et al., 2016). Another alternative σ factor, σ^H^ (*sigH* or SABB_02619), regulates genes related to natural competence for DNA transformation (Morikawa et al., 2012). Maree et al. (2022) demonstrated the occurrence of transformation in biofilm and showed that advantageous conditions for biofilm formation increase the transformation efficiency, identifying SrrA, SigH, and ComK as essential competency regulators (Maree et al., 2022). SigH also regulates the integration and excision of prophages and stability of the lysogenic state, playing an important role in horizontal gene transfer (HGT) (Tao et al., 2010). Last, the extracytoplasmic function (ECF) σ^S^ factor (*sigS* or SABB_01901) is involved in DNA repair response, cell wall stress, and amino acid starvation (Shaw et al., 2008; Miller et al., 2012). We did not include the σ factors in the Bmb9393 GRN but we reconstructed the σ^B^ and σ^H^ regulons due to their importance in the biofilm formation (Supplementary Table S2).

### 3.2. Gene regulatory network of the Bmb9393 strain

The initial step of the reconstruction consisted of the comparative propagation of the GRN of *S. aureus* strain N315 (Ravcheev et al., 2011). The N315 GRN comprises 49 regulons listed in the RegPrecise database (http://regprecise.lbl.gov/). Except for PdxR, YdfD/YisV, and RbsR2, the remaining 46 TFs were orthologous to Bmb9393 genes. For each orthologous TF, we propagated all target genes that were orthologous to Bmb9393 genes. It is important to mention that we choose not to include in the GRN truncated TFs annotated as pseudogenes. It was the case of the repressor MecI which has a truncated gene (mecI*) in the genome of Bmb9393, and appears to be conserved and non-functional in the ST239-SCC*mec*III lineage (Harris et al., 2010; Oliveira and de Lencastre, 2011).

After the propagation, we performed an *in silico* validation, and the expansion of the draft GRN based on searching for TFBSs from orthologous TFs in the upstream region of Bmb9393 coding sequences. Using the list of TFBSs recovered from *S. aureus* TF orthologs, we validated the presence of propagated target genes. In addition, we included three new regulons, CsoR, SarA, and MgrA identified by our approach. From the 72 *B. subtilis* orthologous TFs to Bmb9393, we retrieved information about 40 genes, of which 35 were already in the Bmb9393 GRN. We analyzed the variability level between the TFBSs of both species according to the classification applied by Ravcheev et al. (2011), and identified 13 TFBSs classified as substantially different from the perspective motifs of *B. subtillis* (CggR, MntR, PurR, CsoR, CzrA, BraR, CstR, GraR, YtrA, HisR, GlvR, and NreC e RbsR), 7 as moderately different (ArgR, BglR/YydK, FapR, FruR, MtlR, and TreR e Zur), and 15 TFBSs were well conserved or only slightly variable (BirA, CcpA, CodY, CtsR, CymR, Fur, GltC, GlnR, GntR, HrcA, LexA, MurR, NrdR, and PerR e Rex) (Ravcheev et al., 2011). After the exclusion of the 13 TFBSs substantially different, the searching in Bmb9393 resulted in the addition of 12 new targets for CcpA (3 of them were experimentally validated, *man*PA, *drp35*, and *metK*), 2 for CodY (*nrd*IEF, and *vfr*BA), and one for Fur (SABB_01577). The moderately different TFBSs (2 to 4 non-conserved bases) did not return any potential target gene in Bmb9393 corroborating the results obtained by Ravcheev et al. (2011) that TFBSs are species-specific (Ravcheev et al., 2011). The remaining five orthologous TFs (CcpE, ComK, GbsR, MhqR, and SrrA, apart from SigB-Supplementary Table S2) were included as new regulons after the search in the intergenic regions of Bmb9393 for TFBSs recovered from *B. subtilis* to identify target genes, followed by manual curation of the results. The last step involved the GRN enrichment, based on searching in the literature for new TFs that were not submitted to any specific regulatory database. We retrieved a list of 51 TFs of which 23 were excluded because they are already in the draft GRN, do not have orthologs in Bmb9393, or were not characterized. Of the remaining 28, we identified TFBSs for 11 TFs (BraR, CstR, GraR, HypR, Rsp, SarR, WalR, SarT, QsrR, ArlR, and GbaA) and σ^H^, and used those TFBSs to search in the upstream region of Bmb9393 coding sequences to identify new potential target genes.

**Figure 2:**
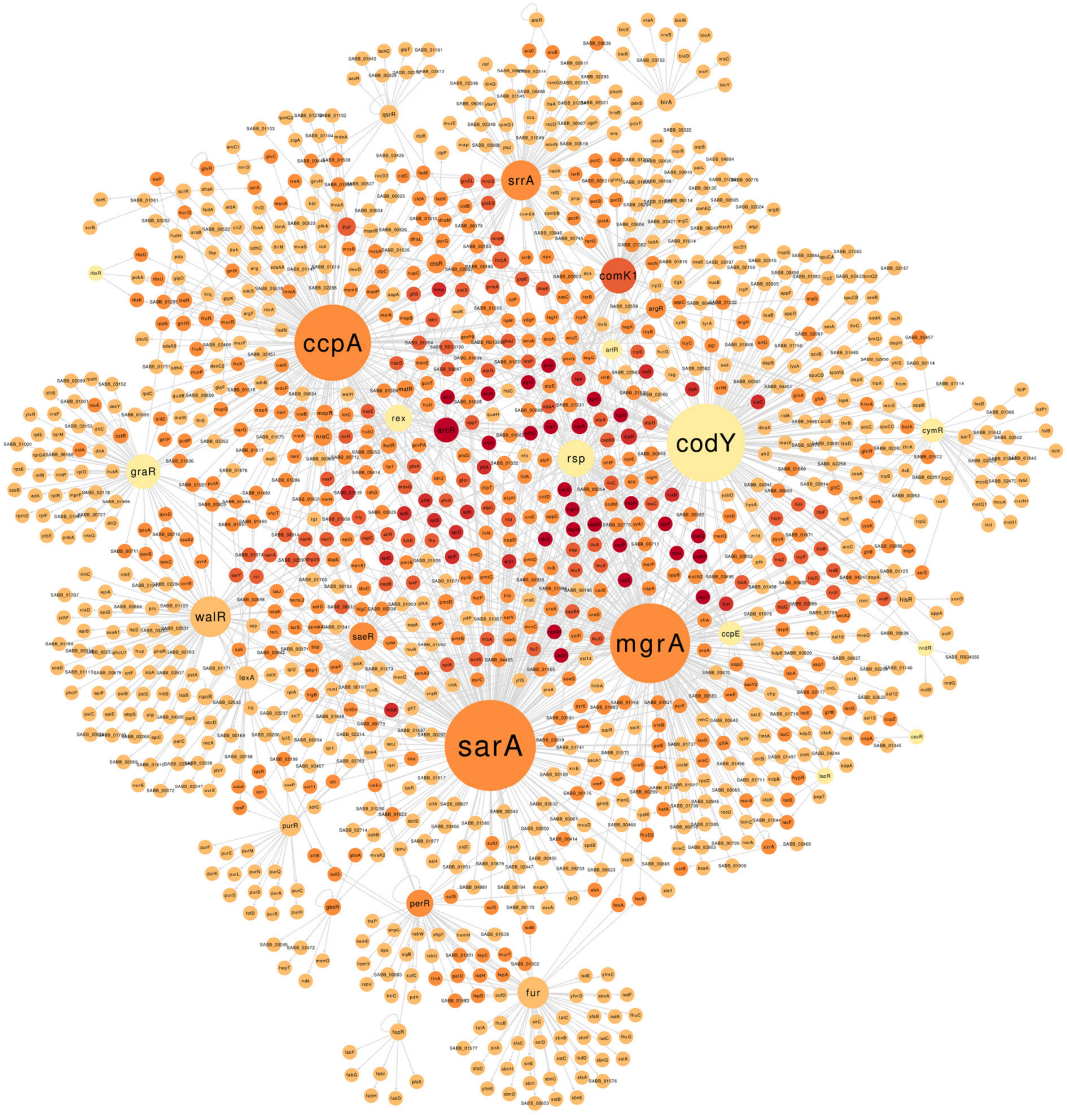
The largest connected component of the GRN Bmb9393. The size of the nodes is proportional to the outdegree values (number of edges leaving the node), with darker node colors indicating higher indegree (number of edges arriving at the node). The GRN of the Bmb9393 with all connected components can be found in Supplementary Figure S2.

The final GRN comprises 1,803 interactions between 64 TFs and 1,151 non-redundant target genes (Supplementary Figure S2 and Supplementary Table S3 available on https://github.com/maiolivei/GRN_Bmb9393/blob/main/Supplementary_Table_S3.xlsx). Figure 2 shows the main connected component of the Bmb9393 GRN, where we highlighted the regulons: SarA with 241 target genes, MgrA with 206, and CodY with 200 followed by CcpA with 195 target genes. SarA (SABB_00667) is an important TF that acts together with Agr as the known *sarA*|*agr* regulatory system, the principal regulator of the expression of genes involved in virulence. The system is related to the transition from exponential to post-exponential growth phase when the repression of adhesin biosynthesis and activation of exoprotein production occurs (Blevins et al., 2002; Jenul and Horswill, 2019; Oriol et al., 2021). SarA has a positive and/or negative effect on 241 target genes, including genes related to biofilm formation and several virulence factors such as adhesin, capsule clusters, leucotoxins, and proteases.

The second largest regulon corresponds to the pleiotropic dual transcriptional regulator MgrA (SABB_00733), which triggers several defensive pathways in response to oxidative stress in a SigB-dependent manner (Deochand and Grove, 2017). This TF modulates genes involved in several biological processes such as autolysis, biofilm formation, virulence, antibiotic resistance, staphylococcal capsule biosynthesis, and metabolism (Luong et al., 2006; Lei and Lee, 2020). MgrA has a similar expression pattern to *agr* and acts as a negative regulator of the biofilm as it inhibits the expression of adhesins and the autolysis process while activating extracellular enzymes that degrade adhesive matrix components (Luong et al., 2006; Crosby et al., 2016; Jiang et al., 2018). The 206 target genes of the MgrA regulon in Bmb9393 comprise relevant protein classes with experimental evidence such as exotoxins, adhesins, and transporters.

The global regulator CodY (SABB_00251) controls the expression of several genes related to metabolism and virulence in response to the concentration of isoleucine, leucine, valine, and guanosine triphosphate (Waters et al., 2016). Thus, it works as a link between the nutritional condition and virulence acting mostly as a repressor of its target genes (Pohl et al., 2009). Indeed, among the known modes of regulation present in the Bmb9393 GRN for CodY, 150 out of 200 are negative. In addition, the majority of target genes act on the metabolism, transport of amino acid and carbon, and virulence both indirectly by the repression of *agrB* and *saeR*, and directly through the binding on the promoter region of capsule-related genes, biofilm-related *ica* operon, and protease-encoding genes such as *aur*.

The fourth largest regulon belongs to CcpA (SABB_01861), the main regulator of the carbon catabolite repression phenomenon, where the expression of genes related to the consumption of different carbon sources is repressed by the presence of glucose or another preferred carbon source (Görke and Stülke, 2008). CcpA regulates several central metabolism pathways, amino acid biosynthesis, and virulence in a glucose-dependent and independent manner (Seidl et al., 2006, 2009; Troitzsch et al., 2021). In the Bmb9393 GRN, CcpA acts on the activation and repression of 195 target genes, highlighting the operons for consumption of alternative carbon sources, and operons important for biofilm formation such as *cid*ABC which is responsible for the cell lysis and eDNA release, an essential structural component of biofilm (Rice et al., 2007).

### 3.3. Comparison between the Bmb9393 and N315 GRNs

**Figure 3:**
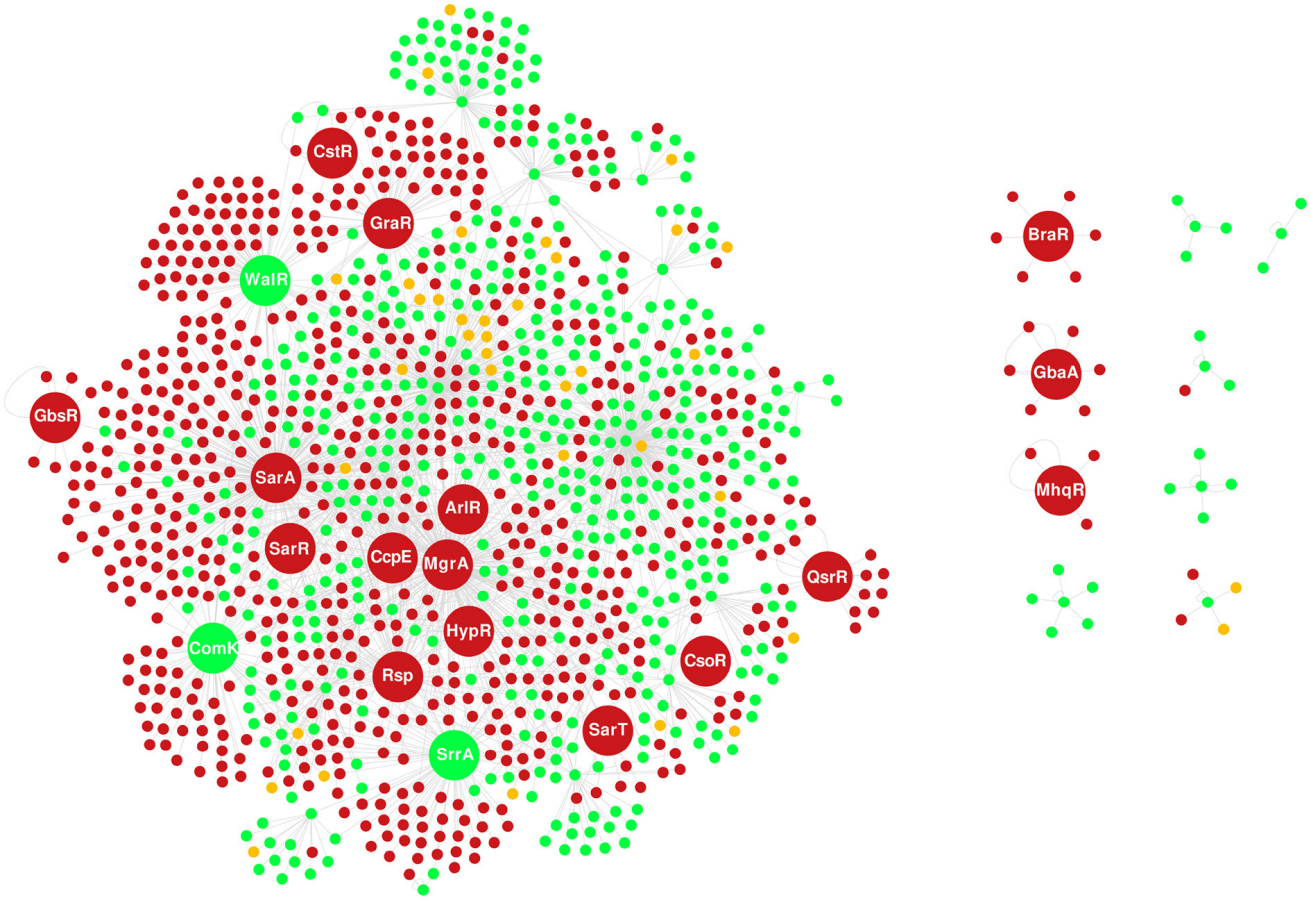
Overlap of the GRNs of Bmb9393 and N315. The nodes in green represent TFs and target genes shared by both strains, the nodes in red correspond to those specific to Bmb9393, and the nodes in yellow are unique to N315. The nodes with larger sizes correspond to the TFs incorporated from the GRN of Bmb9393, where the three shared nodes (ComK, SrrA, and WalR) belonged to the GRN of N315 only as targets for other regulons.

To the best of our knowledge, the reconstruction performed by Ravcheev et al. (2011) is the only *S. aureus* GRN published up to date. The N315 GRN comprises 49 TFs and the non-redundant set of 548 target genes. The overlap of N315 and Bmb9393 GRNs showed that the major variability resides in the target genes of shared global regulators, mainly CodY, CcpA, and Fur. The global TFs recognize a larger number of less conserved TFBSs, showing more flexibility than local TFs that bind with high affinity to one or few TFBSs (Balleza et al., 2009). It is important to mention that we choose to map the orthologous genes between Bmb9393 and N315 to avoid annotation bias. Of the 665 interactions present in the N315 GRN, we found 620 with orthologous genes to Bmb9393. Thus, the N315 GRN contains 45 non-shared interactions with Bmb9393, apart from other differences shown in Figure 3. In addition, we found in Figure 3 the 19 new regulons included in the Bmb9393 GRN, of which 11 were obtained from the literature (ArlR, BraR, HypR, Rsp, SarT, SarR, CstR, WalR, GraR, QsrR, and GbaA), whereas eight were obtained from the expansion methodology (CcpE, ComK1, CsoR, GbsR, MgrA, MhqR, SarA, and SrrA).

SrrA (SABB_03639) is the response regulator of the SrrAB two-component system (TCS) orthologous to ResDE of *B. subtilis*, essential to the growth in anaerobic conditions controlling the respiratory shift from aerobiosis to anaerobiosis (Nakano et al., 1996). Studies with SrrAB (SrhSR) in *S. aureus* showed its relationship with the anaerobic respiratory shift is given by the activation of genes encoding fermentative enzymes (*ldh* and *adh*) and repression of citric acid cycle genes (*acnA* and *fumC*) (Throup et al., 2001). SrrA also controls the regulation of virulence factors in an *agr*-dependent and independent manner, besides the stimulation of biofilm formation (Pragman et al., 2004; Mashruwala et al., 2017). Among the 80 target genes predicted for SrrA, we highlight the *srrA* itself, *agrB, cidA, spa, adhE*, and *hmp*, all confirmed by the literature. Although not experimentally validated, we also mention the SasC adhesin and the regulator Rbf, both related to biofilm formation (Rice et al., 2007; Cue et al., 2009).

Rps (SABB_01294) is another newly included regulator that plays a role in the repression of biofilm formation by the inhibition of surface proteins while activating secreted proteins such as proteases (Lei et al., 2011). In addition, Rsp is relevant to the gene expression adaptation to acute infection development with severe tissue damage by promoting toxin production and limiting biofilm formation, being the latter a typical mechanism of chronic infections (Li et al., 2015). The Rps target genes were all experimentally validated with emphasis on those related to virulence, for instance, *agrB, hld, icaA, fnbA, fnbB*, and the cluster of capsule production.

Last, we highlight WalR (SABB_03186), the response regulator of WalKR TCS (YycG/YycF) that activates autolysin biosynthesis and biofilm formation. The WalKR system is a potential therapeutic due to its essentiality in the Firmicutes phylum, being specific to Gram-positive bacteria with low GC content. In *S. aureus*, apart from the effect on cell wall metabolism, WalKR is important to virulence and antimicrobial resistance, especially vancomycin (Delauné et al., 2012; Peng et al., 2017). Despite its importance, the nature of WalKR essentiality remains unclear. From the 82 target genes identified, we highlight those confirmed by the literature related to cell wall degradation and autolysis (*lytM, isaA, sceD*, and *ssaA*), and virulence (*sdrD, ebpS*, and *sak*). The target gene *ltaS* of the WalR regulon in Bmb9393, classified as a hypothetical protein in previous studies, drew our attention as a possible reason for the reported essentiality of the system. It is responsible for synthesizing lipoteichoic acid and required for bacterial growth and cell division, with experimental evidence of transposon library-based methods confirming *ltaS* as essential in *S. aureus* (Gründling and Schneewind, 2007; Chaudhuri et al., 2009; Santiago et al., 2015).

### 3.4. Comparative topological analysis between the Bmb9393 and N315 GRNs

The topological analysis of both networks reinforced the enrichment of Bmb9393 GRN (Table 2). The average network degree is the sum of all interactions (in and out-degrees) divided by the total number of nodes. The increased mean seen in the Bmb9393 GRN reflects the larger number of interactions present in the network. The network density is the ratio between the number of interactions in a network and the total number of possible interactions. Both Bmb9393 and N315 GRNs showed a low-density measure and are considered sparse networks. This is expected due to the biological nature of gene expression regulation where a small percentage of a genome is composed of regulatory genes and not all regulators control all genes (Cai et al., 2013; Koutrouli et al., 2020). However, the density of Bmb9393 GRN showed to be slightly smaller than N315 as a consequence of the higher number of nodes. Network diameter is the maximum number of edges to go through, considering the shortest possible path between two nodes. Unlike, the radius is the minimum distance between two nodes. In turn, the average shortest path is the mean of the shortest paths of all node pairs in the network. Except for radius, all those metrics are slightly higher in the Bmb9393 network once again reflecting the increased number of nodes and edges.

**Table 2:** Topological metrics between the GRNs of Bmb9393 and N315 strains.

**Network Metrics**	**N315 GRN**	**Bmb9393 GRN**
Nodes	563	1,162
Edges	665	1,803
Average network degree	2.36	3.10
Network density	2.09e^−3^	1.33e^−3^
Network diameter	3	4
Network radius	1	1
Average clustering coefficient	0.056	0.068
Connected components	21	10
Average shortest path	1.21	1.69
Average number of neighbors	2.23	3.02
FFL motifs (*Z*-score)	7 (4.03)	54 (2.13)

The average number of neighbors is another topological measure that reflects the enrichment of the network connectivity. A network can be expanded in the number of nodes without necessarily having an increase in the mean number of neighbors. The improved connectivity of Bmb9393 GRN can be seen by the higher expected number of neighbors for a node. Another measure showing this aspect more directly is the number of connected components that decreased considerably in the Bmb9393 GRN. A connected component is a set of nodes connected to each other by a path. The Bmb9393 GRN has only 10 connected components being the larger composed of 1,097 nodes, while the N315 has 21, the larger containing 449 nodes. A cluster is a set of nodes that are more connected with each other than they are with the rest of the network. The clustering coefficient of a node shows the tendency a node has to form a strongly connected group. Although only slightly, the Bmb9393 GRN showed an average clustering coefficient higher than N315 reinforcing the presence of larger connected communities.

Last, we searched the networks for motifs that can be interpreted as repeated patterns of interaction from which networks are composed. The most recurring pattern found in biological networks is the feed-forward loop (FFL). A FFL is composed of three nodes, which, in the context of gene regulatory networks, two of them are TFs and one target gene. The TF “A” regulates the TF “B”, and the target gene “C” is regulated by both TFs. The biological importance of this type of motif is its contribution to fine-tuning gene expression regulation (Mangan and Alon, 2003; Alon, 2007). We found 7 FFL motifs in the N315 GRN in comparison to 54 in the Bmb9393 GRN (Supplementary Figures S3, S4). The FFLs motifs are classified into eight types depending on the mode of regulation that the TFs exert on target gene but because several interactions in both GRNs have an unknown or dual mode of regulation, we could not precisely estimate which type is prevalent. Among those we could identify, coherent type 2 and incoherent type 2 were the most frequent.

**Figure 4:**
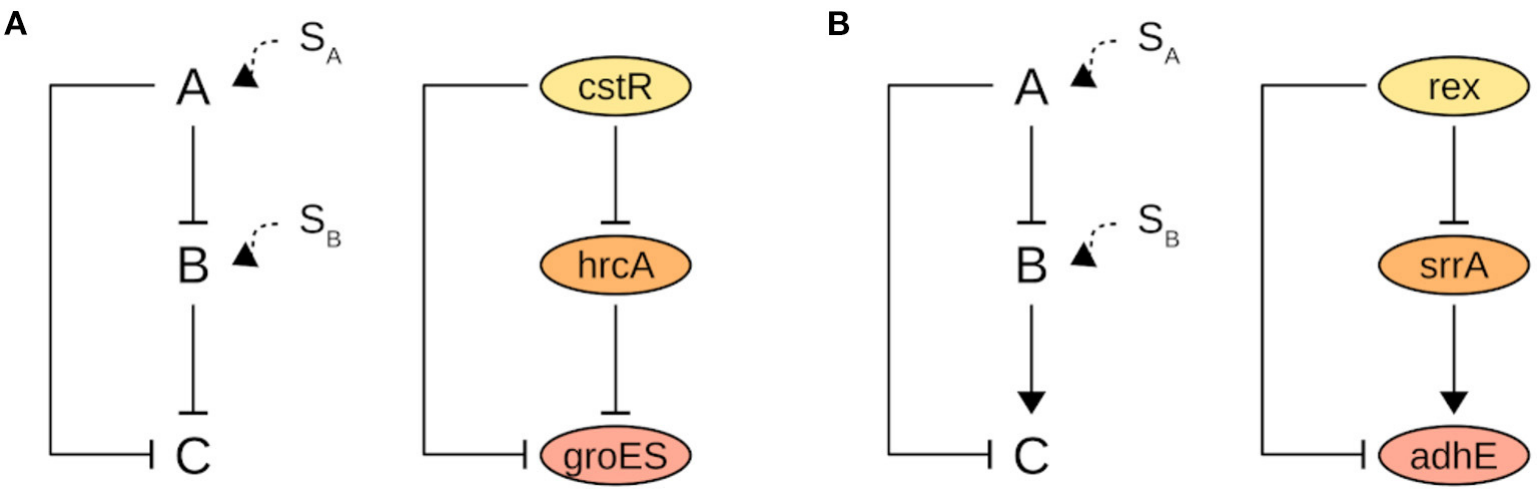
A schematic representation of **(A)** I2-FFL and **(B)** C2-FFL motifs with their respective biological examples in Bmb9393 strain. “C” is the target gene, “A” and “B” are the regulators. SA is the signal for “A” activation, and SB for “B.” Arrows denote activation and blunt-end arrows denote repression.

Overall, the presence of a signal such as an inducer molecule, amino acid modification, or protein interaction activates the TFs which then exert their control upon the target gene. The control effect can be a delayed response in the case of a coherent FFL or a hastened response in the case of an incoherent FFL. The incoherent type 2 FFL (I2-FFL) is formed by two repressors “A” and “B”, and the expression of target “C” requires the absence of the signal for both TFs simultaneously. If only the signal for “A” is present, “C” is repressed but if the signal fades “C” can be readily expressed. If the signal for “B” is present, “C” will be repressed independent of the presence or not of a signal for “A” (Figure 4) (Mangan and Alon, 2003; Alon, 2007). A practical example is the I2-FFL formed by the regulators CstR and HrcA, and the target *groES*. GroES is a chaperone protein that is expressed in response to heat shock stress. Indeed, Chastanet et al. (2003) demonstrated that both regulators act together to maintain the expression level of *groES* under control in the absence of stress (Figure 4A). The target genes composing the other I2-FFLs found are also related to some type of stress response.

The coherent type 2 FFL (C2-FFL) is formed by one repressor “A” and one activator “B”. If “A” and “B” affect “C” expression independently, in the presence of a signal for “A” the repression of C will be delayed. Otherwise, if “A” and “B” affect “C” expression jointly, in the absence of a signal for “A” the activation of “C” will be delayed. A biological example of C2-FFL is the triad Rex, SrrA, and *adhE* (Figure 4B). AdhE is a fermentative enzyme induced under anaerobic conditions. Rex is inactivated when the NAD+/NADH ratio decreases leading to de-repression of *adhE*. However, (Pagels et al., 2010) demonstrated that expression of *adhE* is only strongly upregulated in the Rex mutant in anaerobiosis, suggesting that an additional mechanism is required to fully induce *adhE* expression. Indeed, the activation of *adhE* is also dependent on SrrA which is also induced under oxygen limitation (Kinkel et al., 2013).

Incoherent FFLs are useful when a speedy response is required, and coherent FFLs when a specific response should only occur in the dependency of a persistent signal. Based on this assumption, motif detection can be useful to draw the attention of researchers to target genes composing the FFL triads that are annotated as hypothetical proteins to investigate their functions.

### 3.5. Assessment of genes associated with the biofilm in diverse backgrounds

Biofilm formation is a complex process with intricate regulation, and its composition is highly influenced by the genetic background of strains and environmental signals. To gain insights into the biofilm formation process from a regulatory point of view obtained from diverse backgrounds, we obtained two non-redundant sets of genes with altered expression in the biofilm: orthologous set of non-redundant DEGs from microarray experiments with 1,061 members, and orthologous set of non-redundant DEGs with 1,310 genes obtained from bulk RNA-seq. The overlap of the two sets revealed 576 shared genes in addition to 485 unique genes from the microarray data and 734 specific to the bulk RNA-seq. Then, we merged both shared and unique genes into a non-redundant set with 1,794 proteins referred to as DEGs in biofilm (Supplementary Table S4 available on https://github.com/maiolivei/GRN_Bmb9393/blob/main/Supplementary_Table_S4.xlsx).

**Figure 5:**
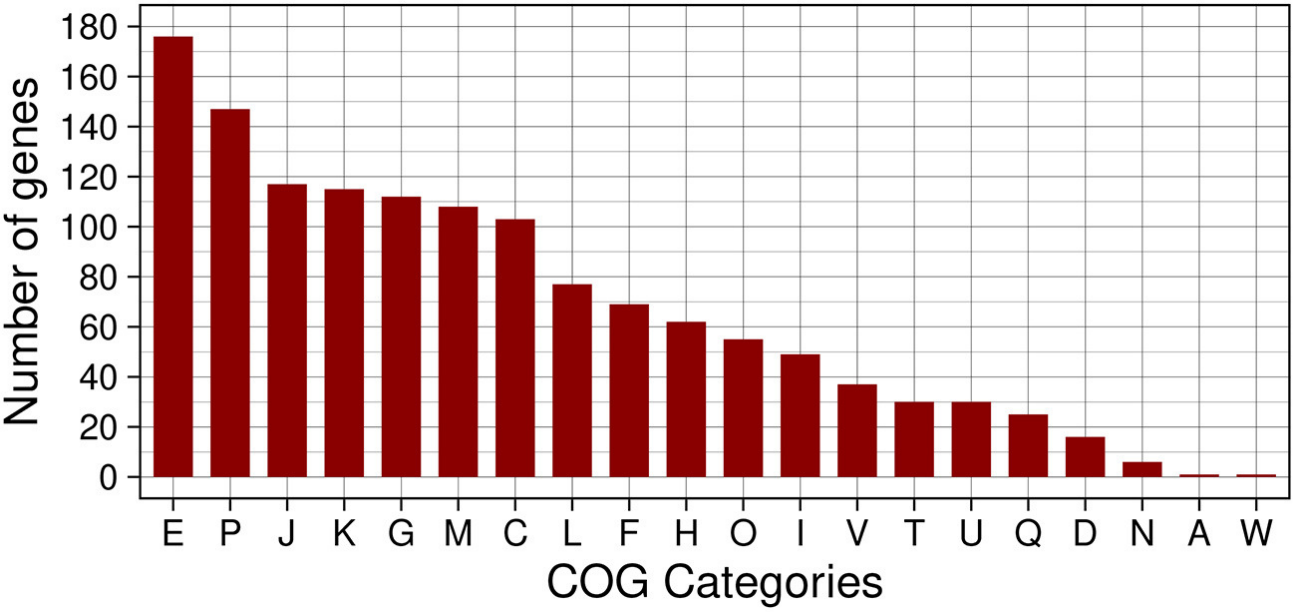
Histogram of COG functional classes of genes with altered expression in the biofilm in S. aureus. The COGs represented in the figure are: E → amino acid transport and metabolism, P → inorganic ion transport and metabolism, J → translation, ribosomal structure and biogenesis, K → transcription, G → carbohydrate transport and metabolism, M → cell wall/membrane/envelope biogenesis, C → energy production and conversion, L → replication, recombination and repair, F → nucleotide transport and metabolism, H → coenzyme transport and metabolism, O → posttranslational modification, protein turnover, chaperones, I → lipid transport and metabolism, V → defense mechanisms, T → signal transduction mechanisms, U → intracellular trafficking, secretion, and vesicular transport, Q → secondary metabolites biosynthesis, transport and catabolism, D → cell cycle control, cell division, chromosome partitioning, N → cell motility, A → RNA processing and modification, and W → extracellular structures.

The functional characterization of the 1,794 proteins into COG categories, after removal of the S category assigned as unknown functions, resulted in the distribution shown in Figure 5. The most representative categories in the set of genes with altered expression in the biofilm were: E for amino acid transport and metabolism, P for inorganic ion transport and metabolism, J for translation, ribosomal structure and biogenesis, K for transcription, and G for carbohydrate transport and metabolism. Indeed, categories E and G encompass the function of target genes regulated by CodY and CcpA that respond to environmental condition variations by modulating the expression of metabolic and virulence genes. Furthermore, the over-representation of the categories K with a large diversity of transcriptional regulators, J with all the apparatus for protein translation, and P with transport systems for several essential metabolites is consistent with the nutritional gradient in the deeper biofilm layers. The chemical heterogeneity resulting from the bacterial metabolic activity and the formation of channels in the biofilm layers produces the oxygen, nutrients, and bacterial waste product gradients that require intense regulation to adapt to locally variable conditions throughout the biofilm formation process (Stewart and Franklin, 2008). Analysis of the ontology for the set of 1,794 proteins with altered expression in biofilm resulted in 2,504 GO terms with 24,718 occurrences. The representative set resulting from REVIGO contains biological processes associated with regulation, amino acid and carbohydrate metabolism, and diverse types of stress responses. The results corroborate those obtained by the COG analysis and highlight the importance of the stress response produced by the microenvironments in the biofilm when the preferential nutrient sources are depleted in the deeper layers and bacteria metabolic shift to alternative sources. One source of accurate information from the literature resides in the KEGG metabolic pathways, so we mapped the set of genes with altered expression in the biofilm to obtain the most prevalent KEGG metabolic pathways and functional hierarchies. This analysis corroborates the previous results where the largest modules of complete pathways were amino acids metabolism followed by carbohydrate metabolism, and cofactors and vitamins. As expected, among the functional hierarchies of BRITE we highlight those related to TFs and TCSs, transport and secretion systems, and antimicrobial resistance genes, in addition to the one related to enzymes with the highest number of proteins.

After the functional annotation of DEGs in biofilm, we identified the presence of classes of proteins considered essential for biofilm formation in the literature, especially for *ica*-independent biofilm, as it is multifactorial and poorly understood. The first class comprises the surface proteins involved in the bacteria-host matrix interactions during colonization such as adhesion, invasion, and immune system evasion processes (Foster et al., 2014). Among these, fibronectin (Fn)-binding proteins A and B (FnBPA and FnBPB) are fundamental factors for *ica*-independent biofilm by enhancing bacterial aggregation and promoting biofilm maturation (O'Neill et al., 2007; Shanks et al., 2008). The FnBPA is present in DEGs in biofilm along with other surface proteins relevant for different phases of biofilm formation, specially in the early to mature stages such as the multifunctional adhesin SpA, SasG, members of the Sdr family (SdrC, SdrD, and SdrE), fibrinogen-binding protein ClfA, proteins of the Isd system (IsdA, IsdB, and IsdC), secreted adhesins Emp and Eap, the elastin-binding protein EbpS and the non-protein adhesin PIA, the fundamental *ica*-dependent biofilm mediator. The absence of some surface-bound proteins in DEGs in biofilm may be explained by the different conditions of the transcriptomes or by the variation in *S. aureus* genomes since FnBPB and ClfB belong to a group that was absent in several strains (McCarthy and Lindsay, 2010). The second class is composed of secreted proteins that modulate biofilm integrity, where proteases are involved in dispersal from the mature biofilm and degradation of the *ica*-independent biofilm matrix (Mootz et al., 2013; Loughran et al., 2014). Indeed, the set of DEGs in biofilm contains all the protease operons and their respective inhibitors such as the serine proteases SspA and the SplA-F operon, the cysteine proteases ScpA and SspB, the metalloprotease Aur, and the intracellular inhibitors of the cysteine proteases ScpB and SspC (Mootz et al., 2013). Another secreted protein with altered expression in the biofilm was α-hemolysin (*hly*/*hla*) which contributes to the initial attachment steps and intercellular adhesion (Caiazza and O'Toole, 2003). The last relevant group for the biofilm is the non-specific cytoplasmic proteins released and incorporated into the biofilm matrix during autolysis. Autolysis is mediated by hydrolases with emphasis on Atl which is essential to the early stages of biofilm by promoting primary adhesion to surfaces and is present in the set of DEGs in biofilm (Houston et al., 2011). Among the cytoplasmic proteins, we can highlight GAPDH and *eno*-encoded enolase as significant in biofilm formation, where both belong to the set of DEGs in biofilm (Foulston et al., 2014).

Finally, we mapped the set of 1,794 proteins with expression altered in the biofilm against the transcription factor repertoire of the Bmb9393 and its GRN to improve understanding of the regulatory circuits that modulate the biofilm formation process under diverse conditions. It is important to mention that all DEG analyses considered only the expression pattern shared among Bmb9393 and the other strains from the selected transcriptomes. Comparison with the transcription factor repertoire resulted in 113 proteins in common with the set of DEGs in biofilm, representing about 62% of the Bmb9393 regulators. As discussed in the functional analysis, high regulatory activity is necessary to respond to the recurrent environmental changes occurring in the heterogeneous microenvironments and well-defined stages of biofilm. The mapping of the 113 regulators against the Bmb9393 GRN revealed 45 shared TFs. Among them, we highlight the inducer WalR and the inhibitor Rsp, besides AgrA, which acts in the quorum-sensing of *S. aureus*, and the response regulator SaeR. The TCS SaeRS acts depending on the strain and environmental conditions to inhibit or induce biofilm formation. In the Newman strain, constitutive expression of SaeRS prevents the production of a robust biofilm, whereas in the USA300 strain is essential for induction of fermentative biofilm (Cue et al., 2015; Mashruwala et al., 2017). Importantly, DEGs in biofilm contains two of the largest regulons of Bmb9393, SarA, and CcpA. A recent paper by Bulock et al. (2022) with the UAMS-1 strain showed that inactivation of CcpA reduced eDNA incorporation into the biofilm matrix (Bulock et al., 2022). Comparison of the 1,794 DEGs in biofilm against the non-redundant set of 1,151 target genes of the GRN resulted in 876 targets with altered expression in the biofilm, of which 370 were regulated by more than one TF and σ factor (Table 3). Growth in biofilm modulated the gene expression of at least one target gene in each regulon of the Bmb9393 GRN, except for BlaI, showing the variability of regulatory response depending on the strain and/or condition.

**Table 3:** TFs with at least one of their target genes altered in the biofilm.

**TF name**	**TF identifier**	**Functional role of the regulon**	**Target genes altered in the biofilm**	**Total target genes of regulon**
*argR*	SABB_02844	Arginine metabolism	14	17
*arsR*	SABB_06187	Arsenic resistance	1	3
*codY*	SABB_00251	Amino acid metabolism	168	200
*fapR*	SABB_00278	Fatty acid biosynthesis	3	8
*gapR*	SABB_00821	Glycolysis	1	6
*graR*	SABB_00706	Virulence/Antimicrobial Resistance	46	62
*lacR*	SABB_01464	Lactose utilization	6	7
*malR*	SABB_00432	Maltose-maltodextrin utilization	5	9
*mepR*	SABB_01515	Multidrug resistance	6	7
*mgrA*	SABB_00733	Virulence	168	206
*mhqR*	SABB_03589	Response to oxidative stress	3	4
*mntR*	SABB_00682	Manganese homeostasis	3	5
*nanR*	SABB_01531	Utilization of sialic acid and N-acetylglucosamine	7	8
*nreC*	SABB_01284	Nitrate/nitrite respiration	11	13
*pmtR*	SABB_03881	Multidrug resistance	4	5
*purR*	SABB_05027	Purine metabolism	19	22
*rex*	SABB_02501	Energy metabolism	32	40
*srrA*	SABB_03639	Response to oxygen availability	54	80

## 4. Conclusions

The regulation of virulence factors in *S. aureus* is the result of a complex and intricate network of regulatory proteins that respond to specific environmental stimuli in infection sites to integrate metabolism and virulence making *S. aureus* a pathogen remarkably adaptive and versatile. Our work described the reconstruction process and structural analysis of the larger *S. aureus* GRN published up to date, comprising 64 TFs connected to 1,151 target genes through 1,803 interactions, of which 610 were new predictions based on the Bmb9393 genome or whose regulatory roles were not yet characterized and await experimental validation. Bmb9393 GRN possesses 19 new regulons compared to N315 related to key cellular functions such as cell wall metabolism, biofilm formation, and virulence. To the best of our knowledge, this is the first GRN inference performed based on the genome of a Brazilian epidemic clone belonging to the ST239-SCC*mec*III lineage with worldwide dispersion that poses relevant phenotypic traits such as the superior ability to accumulate *ica*-independent biofilm. To understand the regulatory impact of bacterial biofilm growth versus planktonic lifestyle, we performed the functional classification of a set of DEGs from several transcriptomes available in the literature and mapped them to the Bmb9393 GRN. The classification of 1,794 proteins showed classes relevant to biofilm formation, and the mapping revealed the extensive impact on regulators and targets, where 45 out of 64 TFs were considered DEGs in any experiment, and regulons had at least one target gene differentially expressed, except the BlaI. Our reconstruction represents the most recent and accurate GRN and serves as an essential starting point for the integration with other biological networks, such as metabolic or cell signaling, and/or experimental data aiming the generation of even more accurate models for this important pathogen.

## Data availability statement

The datasets presented in this study can be found in online repositories. The names of the repository/repositories and accession number(s) can be found in the article/Supplementary material.

## Author contributions

MC performed the identification of the transcription factor repertoire, GRN reconstruction, identification and functional characterization of DEGs in biofilm, and the overlapping of DEGs with the GRN of Bmb9393. EP-R performed the classification into regulatory families. YM developed the text mining pipeline. AN performed the topological analysis and identification of motifs. MC, AN, and MN performed the network curation efforts. MN, EP-R, MS, and AF supervised the work. MC wrote the manuscript with inputs from AN, and critical feedback from YM, MS, AF, EP-R, and MN. All authors have read and agreed to the published version of the manuscript.
