# Supplementary material for: The gene regulatory network of Staphylococcus aureus ST239-SCCmecIII strain Bmb9393 and assessment of genes associated with the biofilm in diverse backgrounds
Source: Front Microbiol. 2023 Jan 10;13:1049819. doi: 10.3389/fmicb.2022.1049819 (PMC9871828; doi:10.3389/fmicb.2022.1049819)
Supplement: Supplementary file 1 [file Data_Sheet_1.PDF]

frontiers

Supplementary Material

1 SUPPLEMENTARY DATA

1.1 Supplementary Methodology

1.1.1 Gene regulatory network reconstruction of Bmb9393 strain

We developed a text mining pipeline to discover evidence of transcription factor regulation in published scientific articles using the Entrez API (Access Programming Interface) in the PubMed NCBI database (Fiorini et al., 2017). The beginning of the pipeline is the search by articles containing primary rules mentions of the *Staphylococcus aureus* organism and the keyword "regulation". The PubMed article identifiers are retrieved by adding the name of each transcription factor as a keyword. With these identifiers, the XML content of the articles is retrieved, parsed, and cleaned to remove unnecessary metadata. The sentences of each article pass by a series of natural language processing steps such as stemming (remove variations of verbal conjugation), removal of stop words (articles, pronouns, and prepositions), and normalization to lowercase (Chowdhary, 2020). The context of the transcription factor is mined from the remaining content based on rule extraction and dependency tree construction to analyze the relation among the sentence terms by proximity. The rule searches for key verbs related to binding or regulation (such as induce, mediate, regulate, block, promote, and repress) connecting the transcription factors and other genes in shortest paths with a maximum distance of 2. A table for each transcription factor provides the results comprising the sentences, article IDs, list of found interacting verbs, and gene names.

1.1.2 Topological analysis and comparison between the GRNs of Bmb9393 and N315 strains

The function `triad_census()` counts all subgraphs formed by three nodes in a graph, which are classified into 16 types according to all possible combinations of edges and directions among them (available at `https://igraph.org/r/html/latest/triad_census.html`). The function returns a vector of length 16, where each position will contain the total counts of each type of subgraph found in the graph. Position 9 of the vector describes the number of identified triads of type '030T' which represents the pattern corresponding to the feed-forward-loop motif. It is worth mentioning that R indexing starts from 1.

1.2 Supplementary Results

The original annotation contained 177 genes whose products were associated with regulation. We compared this set with the compilation of TFs for Bmb9393 available in the P2TF database with 133 predictions divided into two classes: "Other DNA-binding Proteins" that may contain proteins without regulatory functions and "Transcription Factors" subdivided into categories and regulatory families (Ortet et al., 2012). The comparison between data from original annotation and P2TF resulted in 127 shared genes, with six unique to the database (all false positives or without enough evidence to be considered as TF) and 50 found only in annotation, where two TFs were previously misclassified as regulators. P2TF prediction could not identify even experimentally validated TFs such as SarA, so we decided to submit the Bmb9393 genome to the P2RP tool developed in 2013 by the P2TF database group (Barakat et al., 2013). It was the first to enable

the identification and analysis of regulators in sequences provided by users. In Bmb9393, we identified 140 regulatory proteins, of which 11 belong to the "Other DNA-binding Proteins" class. The comparison of the P2PR results and manual annotation showed 133 shared elements, with 44 exclusive to original annotation and the seven specifics to the tool, all of which were false positives. In addition, the comparison between the P2PR and P2TF revealed a shared set of 123 members, 17 specific to the tool and ten exclusives of the database, displaying slight differences in methodologies applied to classify the regulators.

From articles in the literature focused on TFs in *S. aureus* strains, we selected the work by Ibarra et al. (2013), where the set of regulators of the strain USA300-FPR3757 was defined and classified into 36 families, besides the analysis of the orthologs conservation within eubacterial species (Ibarra et al., 2013). Of the 135 TFs from Ibarra et al. (2013), 119 have orthologs in the Bmb9393, and the comparison with the manual annotation resulted in an overlap of 113 TFs, where six are unique to the article and 63 are exclusive to SABIA. Of the six uniques to the set of Ibarra et al. (2013), we included in the Bmb9393 repertoire: CspLA (SABB_02113), the GbsR repressor (SABB_02023), CspA (SABB_06124), CspC (SABB_06091), and an Xre family transcriptional regulator (SABB_02823), all with incomplete annotation previously, except the SABB_02420 that encodes aminopyrimidine aminohydrolase enzyme (Swiss-Prot identifier Q5HEA5). The graph from Figure S1 compares the classification into regulatory families and abundances of the 135 TFs and σ factors of USA300 (Ibarra et al., 2013) with the 180 regulators of Bmb9393.

2 SUPPLEMENTARY TABLES AND FIGURES

2.1 Tables

Table S1: Repertoire of transcription factors and σ factors identified for the strain Bmb9393.

**Locus_tag Bmb9393**	**Gene name**	**Product name**
SABB_00044	*hup*	DNA-binding protein HU
SABB_00059	*birA*	Bifunctional ligase/repressor BirA
SABB_00098	*arlR*	Response regulator ArlR
SABB_00149	*msrR*	Regulatory protein msrR
SABB_00153	*glcT*	Protein GlcT
SABB_00160	*acnA*	Aconitate hydratase
SABB_00170	*lexA*	LexA repressor
SABB_00185	*desR*	Transcriptional regulatory protein desR
SABB_00209	*glpP*	Glycerol uptake operon antiterminator regulatory protein
SABB_00224		XRE family transcriptional regulator
SABB_00229		GntR family HTH-type transcriptional regulator
SABB_00251	*codY*	GTP-sensing transcriptional pleiotropic repressor CodY
SABB_00270		Putative signal recognition particle (SRP) pathway regulator
SABB_00278	*fapR*	Transcription factor FapR
SABB_00422	*fur*	Ferric uptake regulation protein
SABB_00430		AraC family HTH-type transcriptional regulator
SABB_00432	*malR*	HTH-type transcriptional regulator malR
SABB_00474		Ferric uptake regulation protein homolog
SABB_00481	*sigA*	RNA polymerase sigma factor SigA
SABB_00483	*yqfL*	Putative pyruvate, phosphate dikinase regulatory protein
SABB_00484	*ccpN*	Transcriptional repressor CcpN
SABB_00501	*hrcA*	Heat-inducible transcription repressor HrcA
SABB_00542	*cymR*	HTH-type transcriptional regulator CymR
SABB_00572	*ctsR*	Transcriptional regulator CtsR
SABB_00667	*sarA*	Transcriptional regulator SarA
SABB_00682	*mntR*	Putative transcriptional regulator MntR
SABB_00706	*graR*	Response regulator protein GraR
SABB_00719	*ccpE*	Catabolite Control Protein E
SABB_00733	*mgrA*	HTH-type transcriptional regulator mgrA
SABB_00747	*fruR*	HTH-type transcriptional regulator FruR

SABB_00817	*whiA*	Probable cell division protein WhiA
SABB_00821	*cggR*	Central glycolytic genes regulator
SABB_00865		XRE family transcriptional regulator
SABB_00866		XRE family transcriptional regulator
SABB_00944	*citR*	Putative HTH-type transcriptional regulator CitR
SABB_00965	*spxA*	Regulatory protein spx
SABB_00993	*comK1*	Competence protein ComK
SABB_01018		MarR family HTH-type transcriptional regulator
SABB_01062	*potR*	Putative spermidine/putrescine transport regulator PotR
SABB_01081		Regulatory protein ylbF
SABB_01093		XRE family transcriptional regulator
SABB_01096		Putative phage anti-repressor protein
SABB_06110		XRE family transcriptional regulator
SABB_01108	*gbaA*	Glucose-induced biofilm accessory protein A
SABB_01114 (SABB_06306)		TetR family HTH-type transcriptional regulator
SABB_05425	*cidA*	Holin-like protein CidA
SABB_01151		Putative PTS regulator
SABB_01170		MarR family HTH-type transcriptional regulator
SABB_01185	*sarU*	HTH-type transcriptional regulator sarU
SABB_01186	*sarT*	HTH-type transcriptional regulator sarT
SABB_01282		Putative nitrogen regulatory protein A
SABB_01284	*nreC*	Oxygen regulatory protein nreC
SABB_01285		MerR family HTH-type transcriptional regulator
SABB_01289	*sarZ*	HTH-type transcriptional regulator sarZ
SABB_01294	*rsp*	AraC family HTH-type transcriptional regulator Rsp
SABB_01298		TetR family HTH-type transcriptional regulator
SABB_01310		LytTr family HTH-type transcriptional regulator
SABB_01312	*hssR*	Heme response regulator hssR
SABB_01318	*tcaR*	HTH-type transcriptional regulator tcaR
SABB_01321		TetR family HTH-type transcriptional regulator
SABB_01350	*glvR*	HTH-type transcriptional regulator GlvR
SABB_01357		RpiR family HTH-type transcriptional regulator
SABB_01361		HTH-type transcriptional regulator

SABB_01374		AraC family HTH-type transcriptional regulator
SABB_01376	*rsr*	Repressor of sarR
SABB_01377	*sarR*	HTH-type transcriptional regulator sarR
SABB_01404	*sarV*	HTH-type transcriptional regulator sarV
SABB_01406		MarR family HTH-type transcriptional regulator
SABB_01460		MerR family HTH-type transcriptional regulator
SABB_01464	*lacR*	Lactose phosphotransferase system repressor
SABB_01496	*salA*	Iron-sulfur cluster carrier protein
SABB_01515	*mepR*	MarR family HTH-type transcriptional regulator mepR
SABB_01516		PRD family transcriptional antiterminator
SABB_01531	*nanR*	HTH-type transcriptional repressor NanR
SABB_01538		Phage transcriptional regulator
SABB_01579	*rbsR*	Ribose operon repressor
SABB_01586	*bglR*	GntR family HTH-type transcriptional regulator of beta-glucosides utilization
SABB_01591	*lytR*	Sensory transduction protein lytR
SABB_01629	*hptR*	Response regulator protein HptR
SABB_01650	*murR*	HTH-type transcriptional regulator MurR
SABB_01694		XRE family transcriptional regulator
SABB_01706 (SABB_06014)		GntR family transcriptional regulator
SABB_01736		AraC family HTH-type transcriptional regulator
SABB_01745		TetR family HTH-type transcriptional regulator
SABB_01762	*cadC*	Cadmium resistance transcriptional regulatory protein CadC homolog
SABB_01766	*cstR*	Putative regulator YrkD
SABB_01770	*xylR*	Xylose repressor
SABB_01789		Transcriptional regulator
SABB_01812	*nrdR*	Transcriptional repressor NrdR
SABB_01819	*phoP*	Alkaline phosphatase synthesis transcriptional regulatory protein phoP
SABB_01831		Regulatory protein YtoI
SABB_01843	*ezrA*	Septation ring formation regulator EzrA
SABB_01847		Putative osmotic stress regulator
SABB_01861	*ccpA*	Catabolite control protein A
SABB_01887	*rot*	HTH-type transcriptional regulator rot
SABB_01901	*sigS*	RNA polymerase sigma factor sigS
SABB_01966	*xdrA*	XRE like DNA-binding regulator A

SABB_01967	*airR*	anaerobic iron-sulfur cluster-containing redox sensor response regulator protein AirR
SABB_01982	*blaR1*	Regulatory protein BlaR1
SABB_02006		TetR family HTH-type transcriptional regulator
SABB_02035	*braR*	Bacitracin resistance associated BraR
SABB_02037		MarR family transcriptional regulator
SABB_02040	*arcR*	HTH-type transcriptional regulator ArcR
SABB_02045		Arginine repressor homolog
SABB_02075	*icaR*	Biofilm operon icaADBC HTH-type negative transcriptional regulator icaR
SABB_02088	*hisZ*	ATP phosphoribosyltransferase regulatory subunit
SABB_02114		XRE family transcriptional regulator
SABB_02122	*dnaA*	Chromosomal replication initiator protein DnaA
SABB_02141		Two-component system regulatory protein
SABB_02160	*treR*	HTH-type transcriptional regulator TreR
SABB_02165	*gltC*	HTH-type transcriptional regulator GltC
SABB_02225		XRE family transcriptional regulator
SABB_02239		XRE family transcriptional regulator
SABB_02240		Putative uncharacterized sigma factor
SABB_02258		XRE family transcriptional regulator
SABB_02280		HTH-type transcriptional regulator
SABB_02287	*perR*	Peroxide-responsive repressor PerR
SABB_02295		Putative regulator YfhP
SABB_02299	*recX*	Regulatory protein RecX
SABB_02332	*hisR*	Putative histidine repressor
SABB_02385		Putative phage transcriptional regulator
SABB_02394	*sigB*	RNA polymerase sigma-B factor
SABB_02408	*kpdE*	KDP operon transcriptional regulatory protein KdpE
SABB_02470	*czrA*	HTH-type transcriptional repressor CzrA
SABB_02472		Lytic regulatory protein
SABB_02501	*rex*	Redox-sensing transcriptional repressor Rex
SABB_02505	*scrR*	Sucrose operon repressor
SABB_02531		Putative phage anti-repressor protein
SABB_02538	*darA*	Cyclic di-AMP receptor A
SABB_02619	*sigH*	RNA polymerase sigma-H factor
SABB_02630	*sarX*	HTH-type transcriptional regulator SarX
SABB_02666 (SABB_06300)	*gntR*	Gluconate operon transcriptional repressor
SABB_02724	*manR*	Transcriptional regulator ManR

SABB_02759	*mtlR*	Transcriptional regulator MtlR
SABB_02764		Putative phage PVL repressor protein
SABB_02821	*pyrR*	Bifunctional protein PyrR
SABB_02839		Rha family phage regulatory protein
SABB_02844	*argR*	Arginine repressor
SABB_02945		LytR family transcriptional regulator
SABB_02953		Putative phage antirepressor
SABB_02959		TetR family transcriptional regulator
SABB_02973		MerR family HTH-type transcriptional regulator
SABB_03020	*sarY*	HTH-type transcriptional regulator SarY
SABB_03098	*sarS*	HTH-type transcriptional regulator sarS
SABB_03151	*blaI*	Penicillinase repressor
SABB_03186	*walR*	Transcriptional regulatory protein WalR
SABB_03187		Putative two-component system WalR/WalK regulatory protein YycI
SABB_03247 (SABB_06269)	*csoR*	Copper-sensing transcriptional repressor CsoR
SABB_03264	*agrA*	Accessory gene regulator A
SABB_03405 (SABB_06303)		LysR family HTH-type transcriptional regulator YwbI
SABB_03589	*mhqR*	HTH-type transcriptional regulator mhqR
SABB_03639	*srrA*	Transcriptional regulatory protein srrA
SABB_03746	*hypR*	Rrf2-Family Repressor HypR
SABB_03818	*saeR*	Response regulator saeR
SABB_03881 (SABB_06242)	*ytrA*	Putative GntR family HTH-type transcriptional repressor YtrA
SABB_03905	*glnR*	HTH-type transcriptional regulator GlnR
SABB_03943	*qsrR*	Quinone-sensing and response repressor QsrR
SABB_03965	*vraR*	Response regulator protein vraR
SABB_04167		Phage repressor
SABB_04251	*merR*	Mercuric resistance operon regulatory protein
SABB_05027	*purR*	HTH-type transcriptional repressor PurR
SABB_05207	*rbf*	Regulator of biofilm formation
SABB_05240	*hutR*	LysR family HTH-type regulator of histidine utilization
SABB_05403		TetR family transcriptional regulator protein
SABB_00068	*mecI*	Methicillin resistance regulatory protein MecI
SABB_03071 (SABB_06020)		PRD family transcriptional regulator
SABB_06027		Putative HTH-type transcriptional regulator YgzD

SABB_06109		XRE family transcriptional regulator
SABB_06134		XRE family transcriptional regulator
SABB_06135		XRE family transcriptional regulator
SABB_06154	*rinB*	Transcriptional activator rinB
SABB_06163		XRE family transcriptional regulator
SABB_06164		XRE family transcriptional regulator
SABB_06187	*arsR*	Arsenical resistance operon repressor
SABB_06246		Putative transcriptional activator rinB
SABB_02113	*cspLA*	Cold shock-like protein CspLA
SABB_02023	*gbsR*	HTH-type transcriptional repressor GbsR
SABB_02823		Putative Xre family transcriptional regulator
SABB_06124	*cspA*	Cold shock protein CspA
SABB_06091	*cspC*	Cold shock-like protein CspC

Table S2: Description of the regulons associated with the σ factors *sigB* and *sigH* in the Bmb9393. The gray background in the table indicates that the genes constitute an operon, and the gene in bold represents the first of the operon where we find in the upstream region a TFBS for the TF in question.

Sigma Factor	Target	Regulation mode	References
sigB	**SABB_05156**	positive	Interaction confirmed by Bischoff et al. (2004)
sigB	SABB_00728	positive	Interaction confirmed by Bischoff et al. (2004) and operon described in AureoWiki database
sigB	SABB_00727	positive	Interaction confirmed by Bischoff et al. (2004) and operon described in AureoWiki database
sigB	SABB_02912	negative	Interaction confirmed by Bischoff et al. (2004)
sigB	**SABB_03033**	positive	Interaction confirmed by Bischoff et al. (2004)
sigB	SABB_01477	positive	Interaction confirmed by Bischoff et al. (2004) and operon described in AureoWiki database
sigB	*asp23*:SABB_01478	positive	Interaction confirmed by Bischoff et al. (2004) and operon described in AureoWiki database
sigB	***sbnA*:SABB_01724**	unknown	RSAT (no substitution – maximum score)
sigB	*sbnB*:SABB_01723	unknown	Operon described by Dale et al. (2004) and in RegPrecise
sigB	*sbnC*:SABB_01722	unknown	Operon described by Dale et al. (2004) and in RegPrecise
sigB	*sbnD*:SABB_03096	unknown	Operon described by Dale et al. (2004) and in RegPrecise
sigB	*sbnE*:SABB_01720	unknown	Operon described by Dale et al. (2004) and in RegPrecise
sigB	*sbnF*:SABB_03469	unknown	Operon described by Dale et al. (2004) and in RegPrecise
sigB	*sbnG*:SABB_01718	unknown	Operon described by Dale et al. (2004) and in RegPrecise
sigB	*sbnH*:SABB_01717	unknown	Operon described by Dale et al. (2004) and in RegPrecise
sigB	*sbnI*:SABB_01716	unknown	Operon described by Dale et al. (2004) and in RegPrecise
sigB	SABB_03094	unknown	RSAT (no substitution – maximum score)
sigB	***ssb2*:SABB_02265**	unknown	RSAT (no substitution – maximum score)
sigB	*rpsR*:SABB_02264	unknown	Operon described by (Fritsch et al., 2019)

sigB	**SABB_02543**	unknown	RSAT (no substitution – maximum score)
sigB	*rsmI*:SABB_02544	unknown	Operon described in AureoWiki database
sigB	SABB_00721	unknown	RSAT (no substitution – maximum score)
sigB	*cdr*:SABB_00939	unknown	RSAT (no substitution – maximum score)
sigB	***msrB*:SABB_00090**	unknown	RSAT (no substitution – maximum score)
sigB	*crr*:SABB_00091	unknown	Operon described in AureoWiki database
sigB	SABB_00092	unknown	Operon described in AureoWiki database
sigB	*sdcS*:SABB_02341	unknown	RSAT (no substitution – maximum score)
sigB	***atpF*:SABB_02432**	unknown	RSAT (no substitution – maximum score)
sigB	*atpH*:SABB_02431	unknown	Operon described in AureoWiki database
sigB	*atpA*:SABB_02430	unknown	Operon described in AureoWiki database
sigB	*atpG*:SABB_02429	unknown	Operon described in AureoWiki database
sigB	*atpD*:SABB_02428	unknown	Operon described in AureoWiki database
sigB	*atpC*:SABB_02427	unknown	Operon described in AureoWiki database
sigB	SABB_02144	negative	Interaction confirmed by Bischoff et al. (2004)
sigB	**SABB_02246**	positive	Interaction confirmed by Bischoff et al. (2004)
sigB	SABB_02247	positive	Operon described in AureoWiki database
sigB	SABB_02248	positive	Operon described in AureoWiki database
sigB	**SABB_00621**	positive	Interaction confirmed by Bischoff et al. (2004)
sigB	SABB_00622	positive	Interaction confirmed by Bischoff et al. (2004) and operon described in AureoWiki database
sigB	*proP*:SABB_00624	unknown	RSAT (no substitution – maximum score)
sigB	SABB_00725	positive	Interaction confirmed by Bischoff et al. (2004)
sigB	**SABB_05419**	unknown	RSAT (no substitution – maximum score)
sigB	SABB_02905	unknown	Operon described in AureoWiki database
sigB	*hpf*:SABB_00802	unknown	Operon described in AureoWiki database
sigB	SABB_00847	positive	Interaction confirmed by Bischoff et al. (2004)
sigB	SABB_00889	unknown	RSAT (no substitution – maximum score)

sigB	**SABB_00890**	positive	Interaction confirmed by Bischoff et al. (2004)
sigB	SABB_02924	positive	Interaction confirmed by Bischoff et al. (2004) and operon described in AureoWiki database
sigB	SABB_01044	unknown	RSAT (no substitution – maximum score)
sigB	***gcvT*:SABB_00459**	unknown	RSAT (no substitution – maximum score)
sigB	*gcvPA*:SABB_03797	unknown	Operon described in RegPrecise database
sigB	*gcvPB*:SABB_00457	unknown	Operon described in RegPrecise database
sigB	**SABB_01864**	positive	Interaction confirmed by Bischoff et al. (2004)
sigB	SABB_01863	positive	Interaction confirmed by Bischoff et al. (2004) and operon described in AureoWiki database
sigB	***mhqR*:SABB_03589**	unknown	RSAT (no substitution – maximum score)
sigB	*mhqE*:SABB_01164	unknown	Operon described by (Fritsch et al., 2019)
sigB	*mhqD*:SABB_01165	unknown	Operon described by (Fritsch et al., 2019)
sigB	SABB_01162	positive	Interaction confirmed by Bischoff et al. (2004)
sigB	*ridA*:SABB_02552	positive	Interaction confirmed by Bischoff et al. (2004)
sigB	***acpP*:SABB_00274**	unknown	RSAT (no substitution – maximum score)
sigB	*rnc*:SABB_00273	unknown	Operon described in AureoWiki database
sigH	***comGA*:SABB_00466**	positive	Interaction confirmed by (Fagerlund et al. (2014))
sigH	*comGB*:SABB_00465	positive	Interaction confirmed by (Fagerlund et al. (2014)) and operon described in AureoWiki database
sigH	*comGC*:SABB_02848	positive	Interaction confirmed by (Fagerlund et al. (2014)) and operon described in AureoWiki database
sigH	*comGD*:SABB_04962	positive	Interaction confirmed by (Fagerlund et al. (2014)) and operon described in AureoWiki database
sigH	*comGE*:SABB_03576	positive	Interaction confirmed by (Fagerlund et al. (2014)) and operon described in AureoWiki database
sigH	*comGF*:SABB_06168	positive	Interaction confirmed by (Fagerlund et al. (2014)) and operon described in AureoWiki database
sigH	***comEA*:SABB_00508**	positive	Interaction confirmed by (Fagerlund et al. (2014))
sigH	*comEB*:SABB_00507	positive	Interaction confirmed by (Fagerlund et al. (2014)) and operon described in AureoWiki database
sigH	*comEC*:SABB_00506	positive	Interaction confirmed by (Fagerlund et al. (2014)) and operon described in AureoWiki database
sigH	SABB_00505	positive	Interaction confirmed by (Fagerlund et al. (2014)) and operon described in AureoWiki database

2.2 Figures

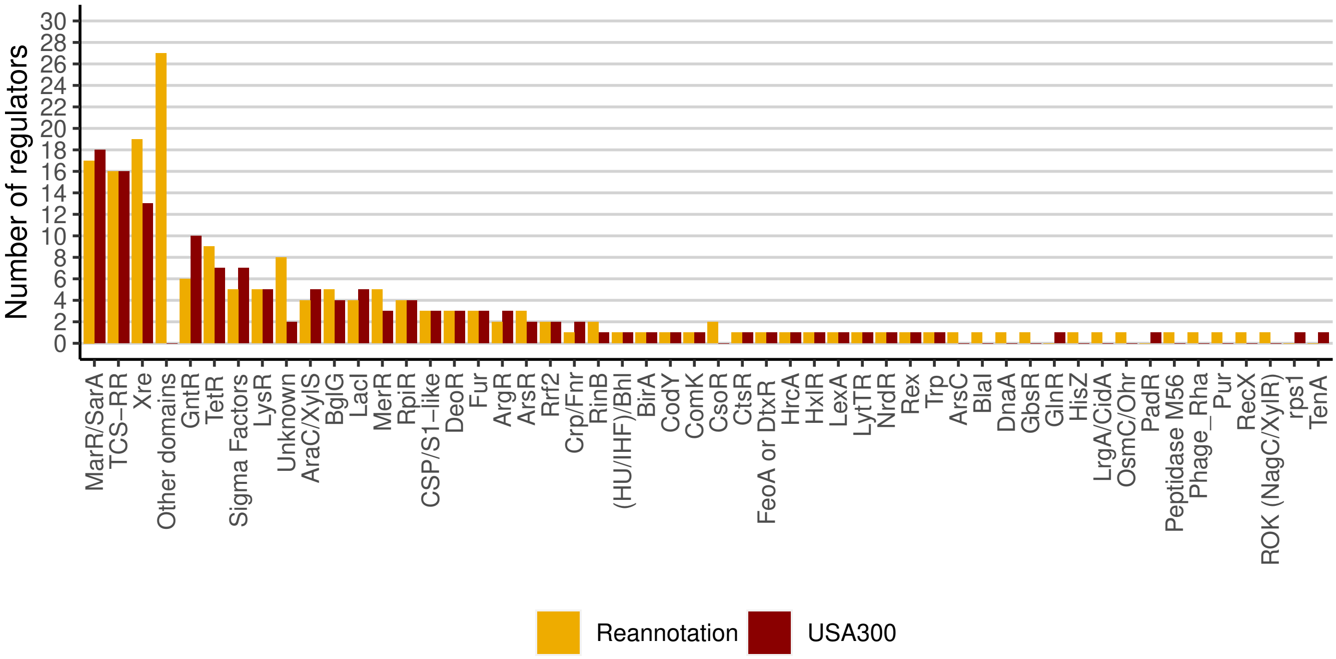

Figure S1: Classification and abundances of regulatory families of Bmb9393 compared to USA300 strain. The yellow columns represent the TFs and σ factors obtained from the re-annotation process, and the red columns represent those identified by Ibarra et al. (2013) to USA300.

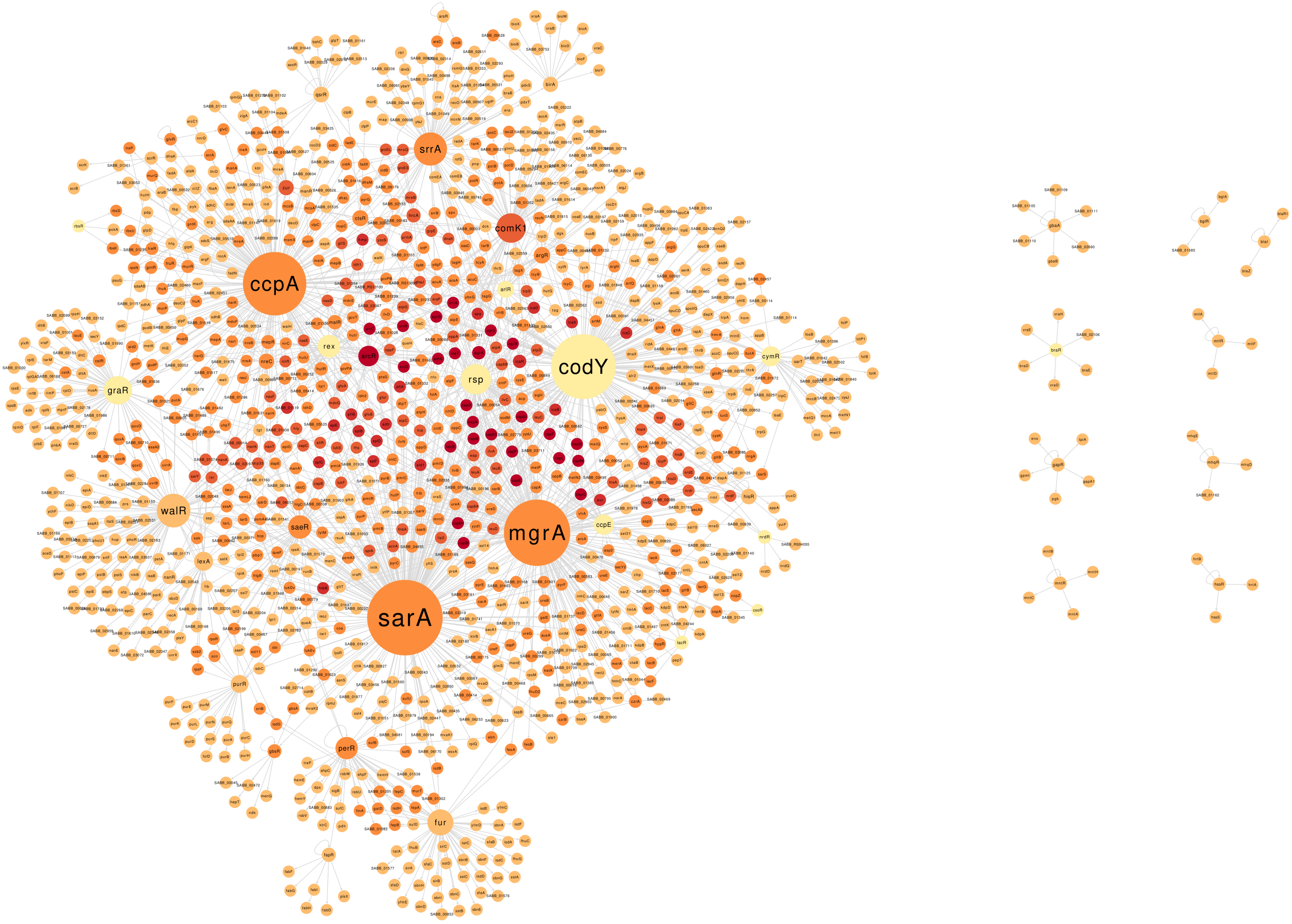

Figure S2: Graphical representation of the GRN Bmb9393. The size of the nodes is proportional to the outdegree values (number of edges leaving the node), with darker node colors indicating higher indegree (number of edges arriving at the node).

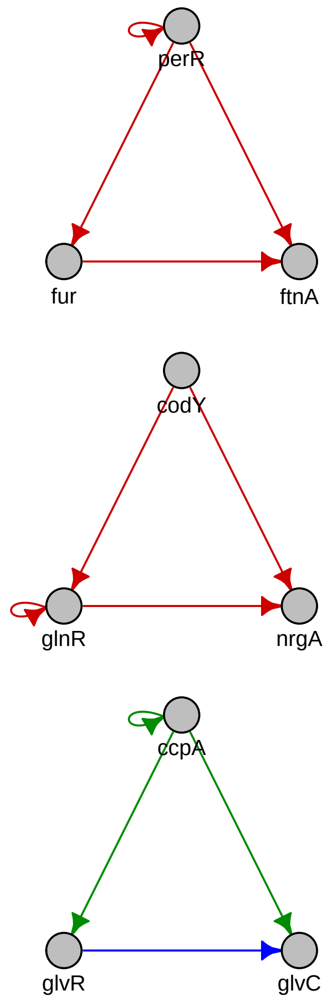

Figure S3: FFL motifs found in the GRN of N315, where the edges colors indicate the regulation mode: red is repression, blue is activation, green is dual, and gray is unknown.

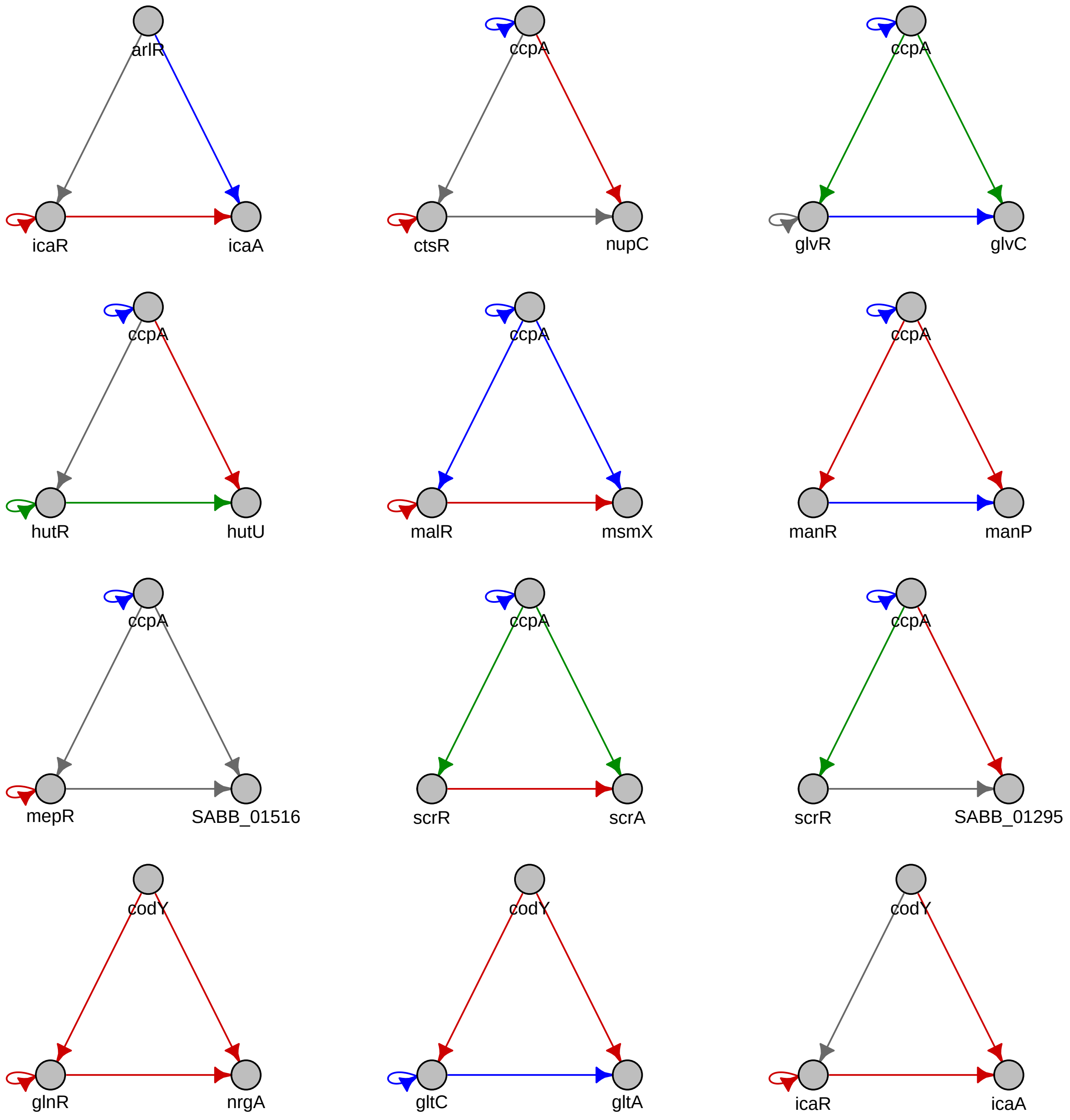
arlR
icaR
icaA
ccpA
ctsR
nupC
ccpA
glvR
glvC
ccpA
hutR
hutU
ccpA
malR
msmX
ccpA
manR
manP
ccpA
mepR
SABB_01516
ccpA
scrR
scrA
ccpA
scrR
SABB_01295
codY
glnR
nrgA
codY
gltC
gltA
codY
icaR
icaA

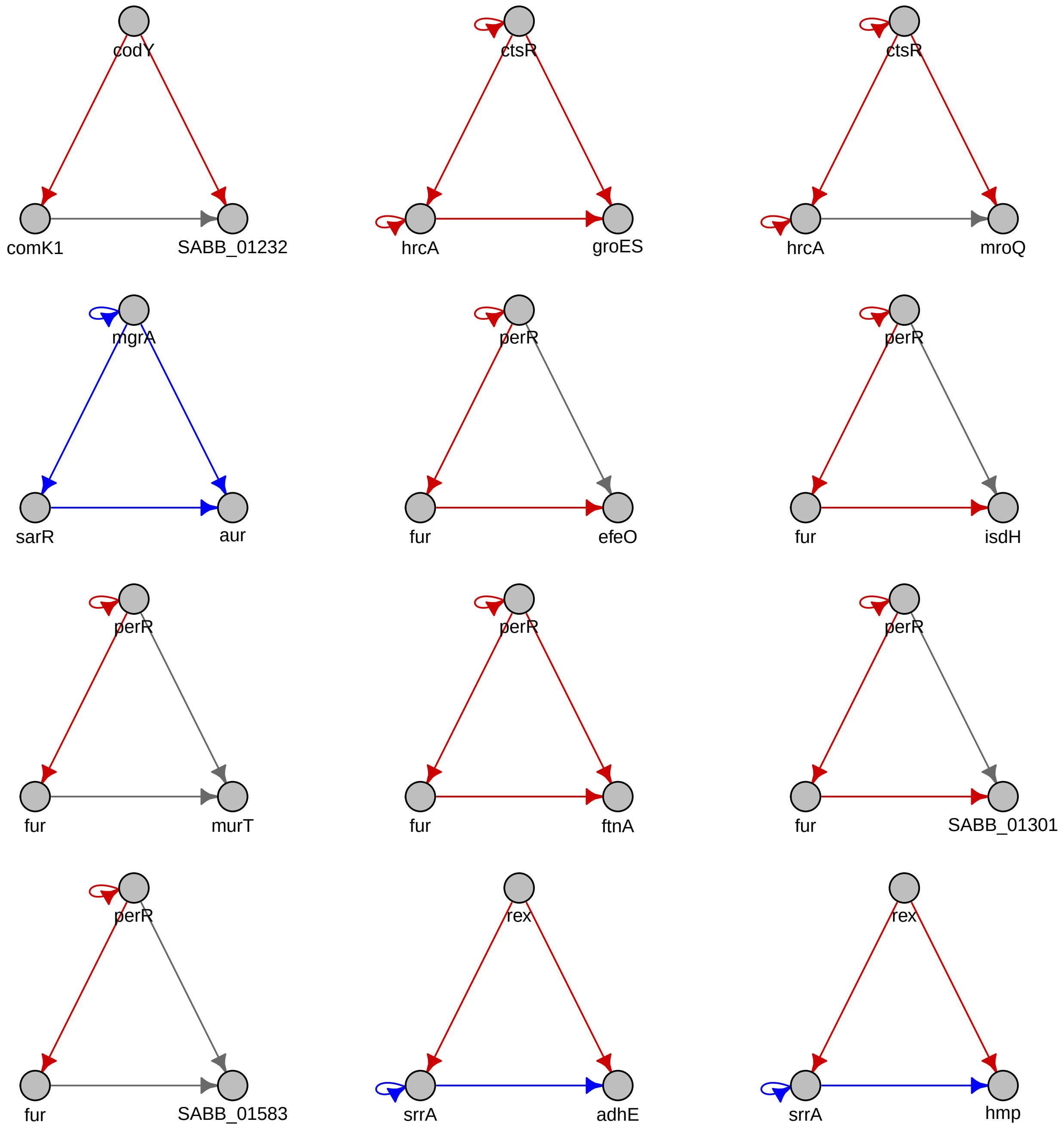
codY
comK1
SABB_01232
ctsR
hrcA
groES
ctsR
hrcA
mroQ
mgrA
sarR
aur
perR
fur
efeO
perR
fur
isdH
perR
fur
murT
perR
fur
ftnA
perR
fur
SABB_01301
perR
fur
SABB_01583
rex
srrA
adhE
rex
srrA
hmp

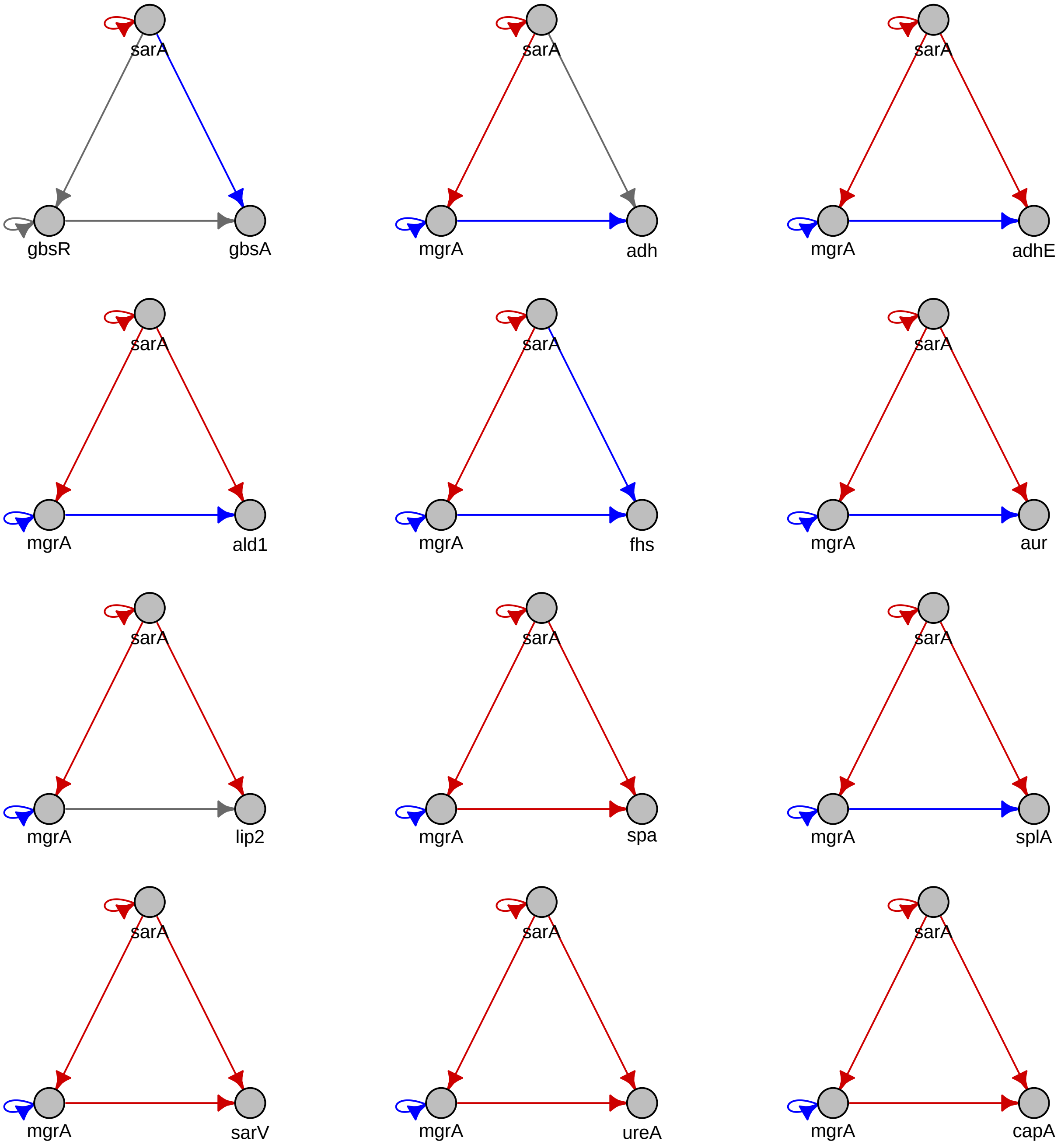
sarA
gbsR
gbsA
sarA
mgrA
adh
sarA
mgrA
adhE
sarA
mgrA
ald1
sarA
mgrA
fhs
sarA
mgrA
aur
sarA
mgrA
lip2
sarA
mgrA
spa
sarA
mgrA
splA
sarA
mgrA
sarV
sarA
mgrA
ureA
sarA
mgrA
capA

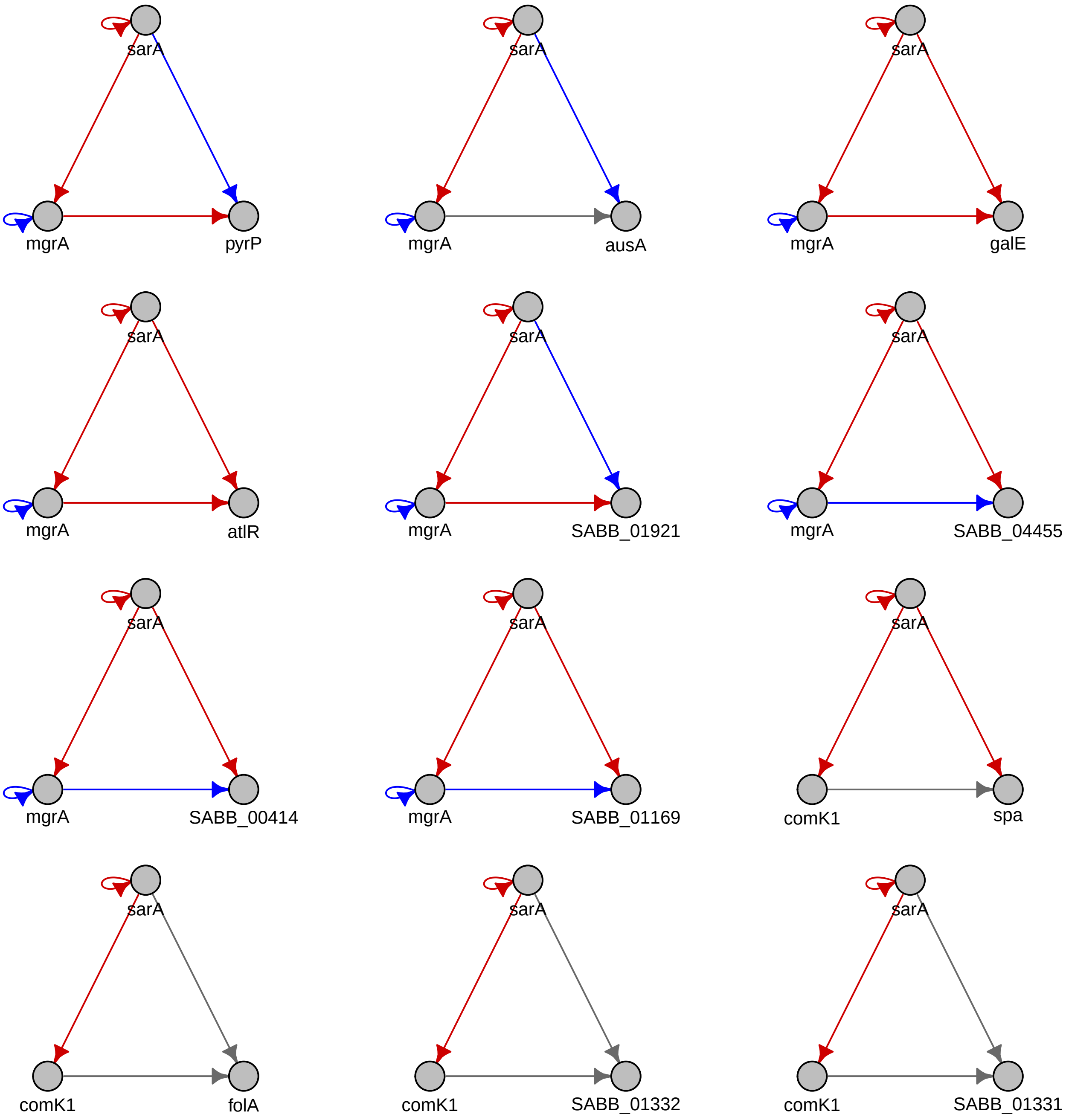
sarA
mgrA
pyrP
sarA
mgrA
ausA
sarA
mgrA
galE
sarA
mgrA
atlR
sarA
mgrA
SABB_01921
sarA
mgrA
SABB_04455
sarA
mgrA
SABB_00414
sarA
mgrA
SABB_01169
sarA
comK1
spa
sarA
comK1
folA
sarA
comK1
SABB_01332
sarA
comK1
SABB_01331

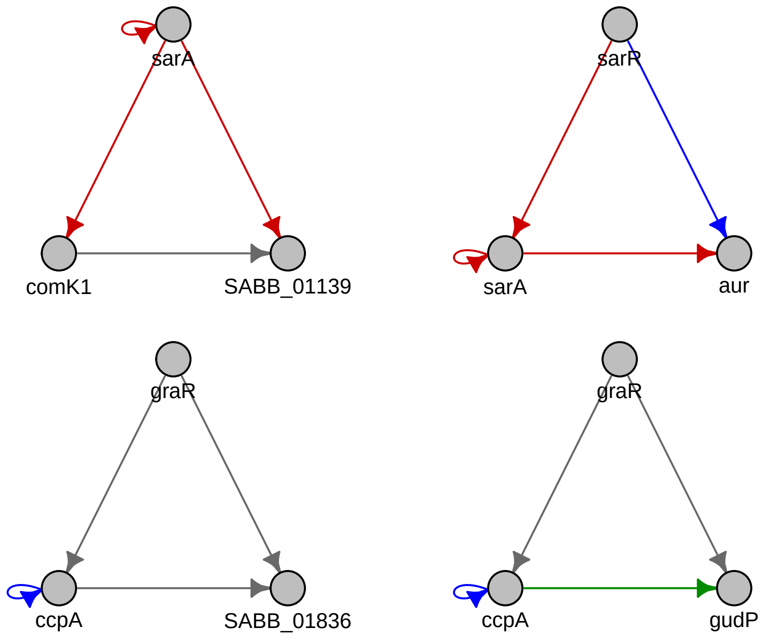

Figure S4: FFL motifs found in the GRN of Bmb9393, where the edges colors indicate the regulation mode: red is repression, blue is activation, green is dual, and gray is unknown.

3 AVAILABILITY OF MATERIALS AND DATA

The gene regulatory network of the Bmb9393 strain and the list of references used to validate its regulatory interactions, just as the list of DEGs (differentially expressed genes) in the biofilm condition with their respective TFs from the GRN of Bmb9393 are available in the online repository which can be reached at `https://github.com/maiolivei/GRN_Bmb9393`.

REFERENCES

Barakat, M., Ortet, P., and Whitworth, D. E. (2013). P2RP: a Web-based framework for the identification and analysis of regulatory proteins in prokaryotic genomes. *BMC Genomics* 14, 269. [PubMed Central:PMC3637814] [DOI:10.1186/1471-2164-14-269] [PubMed:17962296]

Bischoff, M., Dunman, P., Kormanec, J., Macapagal, D., Murphy, E., Mounts, W., et al. (2004). Microarray-based analysis of the Staphylococcus aureus sigmaB regulon. *J Bacteriol* 186, 4085–4099. [PubMed Central:PMC421609] [DOI:10.1128/JB.186.13.4085-4099.2004] [PubMed:9709046]

Chowdhary, K. R. (2020). Natural Language Processing. In *Fundamentals of Artificial Intelligence* (New Delhi: Springer India). 603–649. doi:10.1007/978-81-322-3972-7_19

Dale, S. E., Doherty-Kirby, A., Lajoie, G., and Heinrichs, D. E. (2004). Role of siderophore biosynthesis in virulence of Staphylococcus aureus: identification and characterization of genes involved in production of a siderophore. *Infect Immun* 72, 29–37. [PubMed Central:PMC343950] [DOI:10.1128/IAI.72.1.29-37.2004] [PubMed:8138126]

Fagerlund, A., Granum, P. E., and Håvarstein, L. S. (2014). Staphylococcus aureus competence genes: mapping of the SigH, ComK1 and ComK2 regulons by transcriptome sequencing. *Mol Microbiol* 94, 557–579. [DOI:10.1111/mmi.12767] [PubMed:25155269]

Fiorini, N., Lipman, D. J., and Lu, Z. (2017). Towards PubMed 2.0. *Elife* 6. [PubMed Central:PMC5662282] [DOI:10.7554/eLife.28801] [PubMed:21097890]

Ibarra, J. A., Pérez-Rueda, E., Carroll, R. K., and Shaw, L. N. (2013). Global analysis of transcriptional regulators in Staphylococcus aureus. *BMC Genomics* 14, 126. [PubMed Central:PMC3616918] [DOI:10.1186/1471-2164-14-126] [PubMed:19293374]

Ortet, P., De Luca, G., Whitworth, D. E., and Barakat, M. (2012). P2TF: a comprehensive resource for analysis of prokaryotic transcription factors. *BMC Genomics* 13, 628. [PubMed Central:PMC3532121] [DOI:10.1186/1471-2164-13-628] [PubMed:15034147]
